# Microplastics in freshwater environments across the high altitude region of Gilgit-Baltistan, Pakistan

**DOI:** 10.3389/ftox.2026.1947679

**Published:** 2026-09-22

**Authors:** Lucia Pittura, Sofia Vita, Mattia Resente, Federica Mongera, Adnan Khan, Maisoor Ahmed Nafees, Saif Ud Din, Nelofar Hanif, Giuseppe d’Errico, Alessandro Nardi, Francesco Regoli, Stefania Gorbi

**Affiliations:** 1 Marche Polytechnic University, Ancona, Italy; 2 Scuola Universitaria Superiore Pavia, Pavia, Italy; 3 Department of Animal Sciences, Karakoram International University, Gilgit, Pakistan

**Keywords:** farming, fish, lake, microplastics, mountain, river, sediment, water

## Abstract

Microplastics are increasingly recognized as pervasive contaminants in remote mountain freshwater ecosystems, yet evidence from high-altitude regions remains limited. This study investigated microplastics occurrence and characteristics in surface water, sediments, and fish from the Braldu-Shigar-Skardu freshwater system in Gilgit-Baltistan, northern Pakistan. Samples were collected from remote headwaters, tributaries, main river channels, and the Satpara Lake-Dam system. Fish originated from wild populations, farming facilities, and local markets to provide a broader assessment of microplastic exposure. Matrix-specific extraction protocols were applied, followed by stereomicroscope sorting and polymer identification by µFT-IR spectroscopy. Microplastics were detected in nearly all sites and matrices, with concentrations ranging from 1 to 17 MPs L^-1^ in water, 55–537 MPs kg^-1^ in sediments, and 1–11 MPs individual^-1^ in fish gastrointestinal tracts. Notably, highest frequency of microplastics occurrence was measured in farmed individuals (78%–83%), suggesting that aquaculture-related practices may represent an additional source of microplastics. Wild fish showed the lowest occurrence (25%), whereas those obtained from local markets revealed intermediate values (62%), indicating variable exposure across different pathways. Surface waters were dominated by small fragments and polypropylene, indicative of recent inputs and origin from fragmentation. Sediments showed a broader size distribution, and greater polymer diversity, consistent with their role as long-term sinks. The highest polymer diversity was highlighted in fish, indicating multiple exposure pathways. Spatial patterns did not show a geographical downstream increase, but rather a patchy distribution shaped by local anthropogenic input, hydrological connectivity, and depositional dynamics. Glacier-fed rivers primarily act as transport corridors, whereas low-energy environments such as Satpara Lake function as sinks promoting microplastics accumulation. Overall, the study provides the first integrated multi-compartment baseline for microplastics in Gilgit-Baltistan and supports future monitoring and management actions for these high altitude critical freshwater resources.

## 1 Introduction

Microplastics (MPs), commonly defined as synthetic polymer particles smaller than 5 mm (Frias and Nash, 2019), have emerged as ubiquitous contaminants detected across environmental compartments, establishing microplastic pollution as a global and cross-cutting environmental issue (Yacoub and Han, 2026; Bexeitova et al., 2024; Ahmad et al., 2020; Zhang et al., 2020). The persistence of plastic polymers, the multiplicity of emission sources and complex transport and dispersion dynamics contribute to their widespread occurrence (Mishra et al., 2023), while interactions between abiotic and biotic components can generate interconnected effects across ecosystems (Wang Y. et al., 2023), further enhancing the ecological relevance of microplastics contamination. Moreover, MPs are increasingly recognized as an ecotoxicological concern due to their dual role as physical contaminants and vectors of hazardous chemicals (Li et al., 2024; Pittura et al., 2022).

Studies on microplastics contamination have historically focused on marine ecosystems, while freshwater systems, recognized as key components of the global microplastics cycle (Wagner and Lambert, 2018), remain comparatively under-characterized (Bexeitova et al., 2024). This knowledge gap is particularly relevant because freshwater ecosystems are direct recipients of land-based plastic pollution and major conduits for the transport of MPs from terrestrial environments to the oceans (Guo et al., 2024), also supporting high levels of biodiversity and providing essential resources and ecosystem services (Zhao et al., 2024; Wagner and Lambert, 2018). Particular attention should be directed toward high-altitude and remote mountain freshwater ecosystems. These environments, often difficult to access due to the terrain and extreme climatic conditions, are generally characterized by low population densities and limited human activity, with only a few relatively large population centers. Nonetheless, many mountain regions experience high seasonal tourism, which can markedly increase anthropogenic pressures even in remote areas (Padha et al., 2022).

The presence of microplastics within mountain ecosystems has been documented in both biotic and abiotic matrices, indicating that these environments are not exempt from contamination (Pastorino et al., 2023; Bilal et al., 2023a). Atmospheric transport and fallout, such as wind, rain and snow are considered the primary drivers of MPs deposition in higher altitude ecosystems (Lebepe et al., 2025). Moreover, the inadequate waste management, difficulties in waste disposal, and the lack or poor implementation of sustainable tourism policies, significantly promote the accumulation of plastic debris, the subsequent fragmentation and dispersal, thereby contributing to microplastics pollution in mountain regions (Akdemir et al., 2025; Chauhan and Banerjee, 2025; Pastorino et al., 2023; Villanova et al., 2023). Evidence from the Himalayan region and the Tibetan Plateau further demonstrates the occurrence of MPs across diverse freshwater environments. In the Himalaya, contamination has been reported in major river systems, including the Ganga, Indus, Brahmaputra, Alaknanda, and Kosi, as well as in lakes and glacier-fed systems (Talukdar et al., 2023). A recent assessment of the Hindu Kush Himalayas also documented microplastics across river water, river and lake sediments, lake surface water, and glaciers, highlighting substantial spatial variability and the contribution of both local anthropogenic activities and atmospheric transport (Dar and Gani, 2025). Similarly, studies from the Tibetan Plateau have reported MPs in rivers, lakes, and cryospheric environments, including remote areas, with tourism and long-range atmospheric transport identified as important pathways, while glacier-related processes may contribute to downstream transport (Wang T. et al., 2023; Zhang et al., 2024). Recent observations across rivers and lakes have also highlighted marked spatial variability in MPs abundance and composition, linked to local anthropogenic inputs and environmental transport processes (Yang et al., 2025). Nevertheless, research on microplastics pollution in mountain and high-altitude environments remains geographically limited and fragmented (Lebepe et al., 2025). The logistical challenges of conducting fieldwork in hard-to-reach and harsh environments hamper large-scale assessments while methodological heterogeneity hinders cross-study comparability (Chauhan and Banerjee, 2025). Moreover, limited research infrastructure and funding, particularly in developing countries, restrict systematic monitoring and comprehensive evaluation of microplastics pollution in these fragile ecosystems (Dar and Gani, 2025; Martin et al., 2023; van Emmerik and Schwarz, 2020).

In this context, Gilgit-Baltistan in northern Pakistan represents a particularly relevant case study. The region is renowned for its mountainous landscapes, extensive glaciers, numerous alpine lakes and headwater catchments that sustain freshwater resources of critical ecological and socio-economic importance (Mehboob et al., 2024; Ali, 2023). It also hosts the Central Karakoram National Park (CKNP), Pakistan’s largest protected area, and supports a high diversity of alpine and montane species (Qasim et al., 2024; Ullah et al., 2024). In recent years, Gilgit-Baltistan has become an increasingly popular destination for trekking, hiking, and mountaineering tourism. However, the rapid growth in visitor numbers, combined with challenges in solid waste management, has increased anthropogenic pressures on local ecosystems, contributing to plastic contamination and posing new challenges for the conservation and sustainable management of freshwater resources (Mehboob et al., 2024; Ullah et al., 2024). Despite its environmental and hydrological significance, the region remains largely underrepresented in the literature on microplastics pollution in aquatic systems at national scale (Bilal et al., 2023a; Bilal et al., 2023b; Bilal et al., 2023c; Khan et al., 2022; Hafeez et al., 2023; Qaiser et al., 2023; Bashir and Hashmi, 2022; Irfan et al., 2020). To date, evidence from Gilgit-Baltistan itself is limited to a single investigation of MPs in water and sediments from eight alpine lakes in the Karakoram range (Mehboob et al., 2024), while evidence from freshwater fauna within the broader Indus River basin is restricted to river dolphins stranded in low altitude reaches outside Gilgit-Baltistan (Ali et al., 2025). However, integrated assessments of microplastics across interconnected riverine and lacustrine environments and aquatic biota within the glacier-fed freshwater networks of Gilgit-Baltistan remain lacking.

To address this gap, the present study investigated the occurrence and characteristics of microplastics in surface waters, sediments and fish across the Braldu-Shigar-Skardu freshwater system, part of the upper Indus River drainage network in Gilgit-Baltistan region. The activities were carried out within the framework of a scientific expedition on the environmental status of major watersheds in northern Pakistan, and supported by the EvK2CNR Committee, an organization promoting research and sustainable development in high-mountain regions to protect the environment and support local communities (https://www.evk2cnr.org/). By providing original data on MPs pollution, this work aims to improve the understanding of contamination in high-altitude freshwater ecosystems and to inform future monitoring and management strategies in the region.

## 2 Materials and methods

### 2.1 Study area

Located in the northernmost part of Pakistan, Gilgit-Baltistan represents the highest-elevation region of the country, with an average altitude of approximately 4,500 m above sea level (a.s.l.). The territory encompasses the Karakoram, western Himalayas, and Hindu Kush mountain ranges, which host more than 100 peaks exceeding 7,000 m in elevation, including K2, the world’s second-highest mountain. More than 5,000 glaciers act as critical freshwater reservoirs, sustaining river systems that support ecosystem functioning and provide water for drinking and agriculture purposes for low-land areas (Latif et al., 2026; Khalil, 2021).

Baltoro and Biafo glaciers, within the Karakoram Range, are the largest in the region, together forming the longest continuous glacier systems in the world outside the poles (Qasim et al., 2024). These glaciers feed the Braldu River, which flows westward passing the villages of Askole and Korphe, which are the last permanent settlements before the Karakoram wilderness, thus representing the main gateway for the CKNP and for trekking or mountaineering expeditions to the eight-thousanders, including K2. Downstream Askole and Korphe, the Braldu River joins the Shigar River, which flows southward and enters the Upper Indus River near the city of Skardu, one of the principal urban centers of Gilgit-Baltistan with a population of approximately 113,000. The city and its surrounding settlements have experienced significant growth in recent years, also driven by increasing tourism especially in summer months (Hussain et al., 2023). In addition to the glacier-fed river network, the local water availability around Skardu also depends on Satpara Lake, a natural high-altitude lake located ∼8 km south of the city. The lake is primarily fed by the Satpara Stream, which originates from snow and glaciers melting from the Deosai Plateau while nearby groundwater springs provide a continuous hydrological input to the lake. Since the construction of the Satpara Dam, which led to the expansion of the reservoir, the lake has played a strategic role in the Skardu Valley by supplying drinking water, irrigation water, and hydroelectric energy, while also representing a major tourist attraction and an important site for commercial trout farming in the region (Qasim et al., 2024).

These freshwater resources also support fisheries and small-scale fish farming in the area. Gilgit Baltistan hosts several species of cold-water fish, including native snow carps (*Schizothorax spp*.) and introduced trout species (*Salmo* spp. and *Oncorhynchus* spp.) (Moazzam and Osmany, 2024). Trout is considered the most economically important fish species, occurring both as wild and farmed populations. The latter are used to stock high-altitude rivers and lakes, thereby supporting fishery resources and freshwater biodiversity (Baig et al., 2022).

### 2.2 Sampling of water, sediment and fish

Sampling activities in Gilgit-Baltistan were carried out in June 2024, during the summer peak of tourism and before the onset of monsoon season, which typically extends from July to September (Mehboob et al., 2024). Locations for water, sediment, and fish collection were identified along a downstream continuum from the Braldu to the Shigar and Skardu valleys, with the intention to capture both the hydrological diversity and a range of human activity settings of the area. Accordingly, sampling sites included major and minor rivers, streams, and the Satpara Lake-Dam system across locations spanning from remote and isolated areas to urban centers and popular tourist destinations (Figure 1).

**FIGURE 1:**
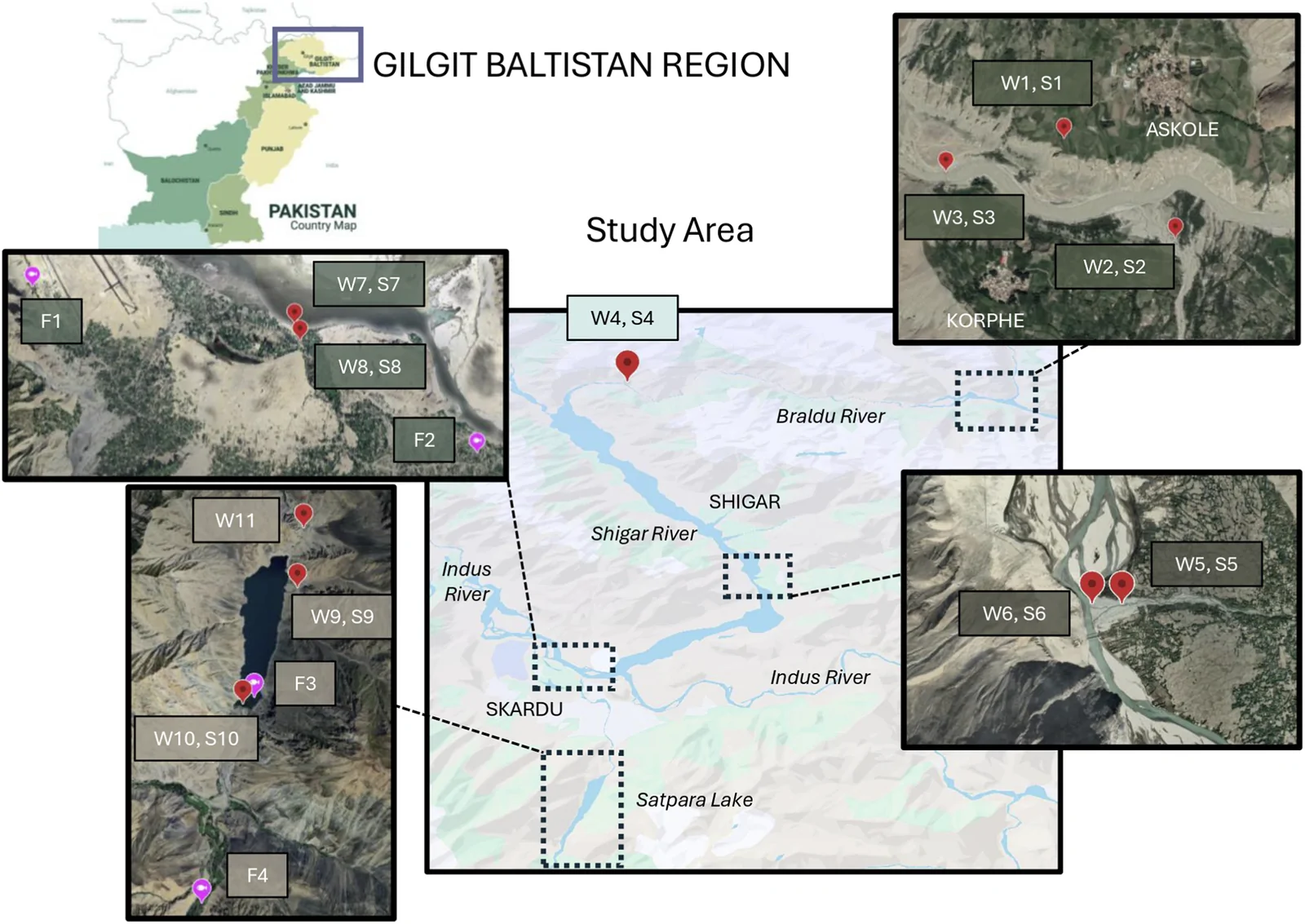
Map showing the sampling locations of surface water (W), sediment (S) and fish (F) across the Braldu, Shigar, and Skardu valleys in the Gilgit-Baltistan region, northern Pakistan.

Specifically, water and sediment samples were collected from 11 shallow wadable locations. Water was sampled at all sites (W1-W11) whereas sediment was sampled at 10 sites (S1-S10), as collection at Satpara Dam was not feasible due to logistical constraints (Table 1). At each site, a single sample was collected for each abiotic matrix, with no replicate samples collected. At each station, 1 L of surface water was collected using Teflon bottles, while sediment was collected using metal spoons and stored in 250 mL Teflon containers.

**TABLE 1:** Information related to the sampling locations of surface water and sediment across the Braldu, Shigar, and Skardu valleys in the Gilgit-Baltistan region, northern Pakistan.

Surface water sample code	Sediment sample code	Sampling site	Geographic coordinates	Altitude (m a.s.l.)
W1	S1	Askole - small stream	35°40′48.15″N 75°48′48.21″E	3020
W2	S2	Korphe - tributary of Braldu river	35°40′35.56″N 75°49′05.79″E	2957
W3	S3	Korphe Bridge - Braldu river (Askole Side)	35°40′44.01″N 75°48′29.63″E	2922
W4	S4	Dassu - Braldu river	35°42′36.64″N 75°32′22.76″E	2425
W5	S5	Shigar - tributary of Shigar river (Bauma Lungma stream)	35°25′27.00″N 75°43′05.99″E	2249
W6	S6	Shigar - Shigar river	35°25′27.02″N 75°42′52.48″E	2242
W7	S7	Skardu - Indus river	35°19′22.97″N 75°35′50.82″E	2216
W8	S8	Skardu - stream	35°19′08.74″N 75°35′56.60″E	2218
W9	S9	Skardu - Satpara lake - spring	35°14′12.60″N 75°38′23.21″E	2733
W10	S10	Skardu - Satpara lake (near Satpara stream inflow)	35°12′45.62″N 75°37′32.77″E	2679
W11	*not available*	Skardu - Satpara lake - dam outlet	35°14′58.29″N 75°38′28.52″E	2598

Fish sampling was restricted to the Skardu territory focusing on the ecologically and commercially relevant species of the area: rainbow trout (*Oncorhynchus mykiss*), brown trout (*Salmo trutta*) and snow trout (*Schizothorax skarduensis*). A total of 31 specimens were collected with the assistance of local fishermen from four sampling sites (samples F1-F4) including wild, farmed and organisms purchased from local fish market in Skardu city (Table 2). The gastrointestinal tract (GIT) was immediately dissected and wrapped in aluminum foil for transport and storage.

**TABLE 2:** Information related to fish sampling restricted to the Skardu Valley in the Gilgit-Baltistan region, northern Pakistan.

Fish sample code	Sampling site	Geographic coordinates	Altitude (m a.s.l.)	Species	Sample size (n)	Fish length (cm)
F1	Skardu city - Fish farm	35°19′55.08″N 75°31′13.42″E	2229	*Oncorhynchus mykiss* *Salmo trutta*	6	25.7 ± 2.2
F2	Skardu city - Fish market	35°17′31.39″N 75°39′02.91″E	2276	*Oncorhynchus mykiss* *Salmo trutta* *Schizothorax skarduensis*	8	26.7 ± 1.8
F3	Satpara lake - wild Fish	35°12′50.43″N 75°37′43.38″E	2680	*Schizothorax skarduensis*	8	26.6 ± 3.5
F4	Satpara lake - Fish farm	35°10′18.00″N 75°36′56.99″E	2904	*Oncorhynchus mykiss* *Salmo trutta*	9	24.6 ± 2

All water, sediment and GIT samples were frozen at −20 °C and maintained at this temperature until processed for microplastics analysis at the laboratory facilities in Italy.

### 2.3 Samples processing

The entire 1 L volume of each water sample was thawed at room temperature and transferred from the Teflon containers used for sampling into glass beakers for processing. To remove suspended organic matter, a 15% hydrogen peroxide (H_2_O_2_) solution was added to each sample at a 1:1 ratio (v/v), following established digestion protocols for MPs extraction in environmental matrices (Pittura et al., 2023). Oxidative treatment was performed in a temperature-controlled oven at 40 °C until no visible organic residues remained, not exceeding 48 h. Subsequently, water samples were vacuum filtered onto cellulose nitrate membranes (8 μm pore size, Ø 47 mm, Sartorius). Filters were recovered in glass Petri dishes and dried at 40 °C overnight prior to microplastics analysis.

Sediment samples were thawed at room temperature and thoroughly homogenized by spatulation before a 25 g aliquot was collected for each site. The aliquots were dried overnight in a temperature-controlled oven at 40 °C. Microplastics were extracted from the dried sediment by centrifugation in a saturated sodium bromide solution (NaBr; density = 1.45 g cm^-3^). Centrifugation is widely employed to enhance density-based microplastics extraction from environmental matrices, including freshwater and marine sediments, soils and sludge (Larrea et al., 2025; Queiroz et al., 2025; Grause et al., 2022; Constant et al., 2021). NaBr was selected because it is non-toxic, non-corrosive, cost-effective and non-destructive to plastic particles while ensuring an efficient recovery across a range of microplastics types (Motiejauskaité and Barčauskaite, 2025; Foglia et al., 2024; Pittura et al., 2021; Liu et al., 2019). Each aliquot was distributed into 50 mL conical centrifuge tubes (Nunc™), with 5 g of sample and 40 mL of NaBr solution in each tube. The mixtures were agitated for 10 min at 250 rpm using an orbital shaker (IKA® KS 260 basic) and subsequently centrifuged at 3,700 rpm for 10 min (Grause et al., 2022). The supernatant was collected in a glass beaker, and the procedure was repeated twice after refilling the tubes with NaBr solution to enhance microplastic recovery (Queiroz et al., 2025; Foglia et al., 2024; Pittura et al., 2021). The final supernatant was then processed as described for the water samples.

The gastrointestinal tracts of fish were processed following the same oxidative digestion and filtration procedures described for water samples. The 15% H_2_O_2_ solution was added in sufficient quantity to fully submerge the tissue and until complete digestion was achieved. Due to the higher complexity of biological tissue, digestion times were longer than those required for water samples and varied according to GIT size, requiring up to 5 days.

### 2.4 Microplastics analysis

All filters resulting from sample filtration, together with the corresponding Petri dishes, were examined under a stereomicroscope (Optika SZM-D) equipped with a digital camera (OptiKam B5) connected to image analysis software (Optika Vision Lite 2.1). During visual inspection, particles resembling plastic were retrieved using tweezers and a mounted needle and transferred onto a new filter support for subsequent chemical analysis by µFT-IR spectroscopy. Suspected microplastics were isolated based on visual, physical, and tactile criteria, including particles resistant to breakage or capable of retaining their shape or deforming without fragmentation when manipulated with tweezers, brightly colored or unnaturally pigmented items, fragments with sharp geometric shapes, and thread-like items (Primpke et al., 2020). All isolated items were subsequently photographed, measured, and further classified according to shape (fragment, film, fiber) and size classes (5–3 mm, 3–1 mm, 1–0.3 mm, 0.3–0.1 mm, and 0.1–0.02 mm), defined considering the upper limit of 5 mm, in accordance with the definition of microplastics (Frias and Nash, 2019), and the lower limit of 20 µm determined by the analytical resolution of the instrument used for chemical characterization. Specifically, a Spotlight 200i FT-IR microscope coupled with a Spectrum Two spectrophotometer and controlled by Spectrum 10 software (PerkinElmer) was used. Infrared spectra were acquired in the mid-infrared region (wavenumber range: 4,000–600 cm^-1^) in micro-ATR mode (µATR), with an optical aperture of 100 × 100 μm. Spectral resolution was set to 4 cm^-1^, and each sample was scanned 16 times to improve signal quality. In addition, multiple background scans were performed during measurements to correct for environmental variations.

Polymer identification was carried out by comparing the obtained IR spectra with reference spectra from commercial libraries (PerkinElmer®), custom-built libraries, and an open-access database (Primpke et al., 2018). Polymer assignment was validated based on match percentages, with values above 70% considered acceptable together with expert evaluation of absorption bands. Items were classified as plastics if they included synthetic polymers and copolymers (Pittura et al., 2022).

### 2.5 Quality assurance and quality control

To minimize contamination during sample processing, cotton laboratory coats and nitrile gloves were worn throughout the analytical procedures and work surfaces were cleaned with ethanol pure grade. All sample preparation and handling activities were carried out in a clean room, with access restricted to a maximum of two operators at the same time. Whenever possible, glass and metal equipment were used and rinsed with ultra-pure water prior to use. Unavoidable plastic materials (e.g., centrifuge tubes) were characterized and excluded from the results if they matched particles detected in samples. Aluminum foils were used to cover equipment and samples to minimize airborne contamination. All solutions were prepared in ultra-pure water and vacuum-filtered through 0.45 μm nitrate acetate membrane before use.

Negative controls were included during both sampling (field blanks) and laboratory processing (laboratory blanks). Field blanks consisted of 250 mL Teflon bottles filled with ultrapure water and left open during sampling activities (N = 11 for abiotic matrices and N = 4 during fish dissection). Upon arrival at the laboratory, field blanks were filtered and analyzed. Laboratory blanks were prepared using 250 mL glass beakers filled with ultrapure water and processed alongside environmental samples according to the same analytical protocol. One laboratory blank was included for each sediment sample (N = 10), while water and GIT samples were processed in batches, resulting in N = 2 and N = 7 laboratory blanks, respectively. Only fibrous particles were detected in both field and laboratory blanks. Subsequent µFT-IR analysis identified all fibers as natural cellulose-based materials, indicating the absence of external plastic contamination during sampling and analyses. As no synthetic particles were detected in the negative controls, no blank correction was applied to the microplastic counts.

### 2.6 Data expression and statistical analysis

Microplastic abundance was expressed as the number of microplastic particles per liter (MPs L^-1^) for water samples and per kilogram of dry sediment (MPs kg^-1^ dry weight, dw) for sediment samples. As only one sample was collected per site for each abiotic matrix (n = 1), statistical comparisons among sites were not performed, and the results are presented descriptively as site-specific values.

For fish samples, microplastics ingestion was expressed as (i) frequency of occurrence calculated as the percentage of individuals containing at least one microplastic relative to the total number of analyzed individuals; (ii) the mean number of microplastics per individual and calculated only for specimens positive to ingestion. Differences in the proportion of fish containing ingested microplastics among the four sampling sites were assessed using Fisher’s exact test (Freeman-Halton extension for 4 × 2 contingency tables). Differences in the number of ingested microplastics among sites were evaluated using the Kruskal-Wallis test due to the non-normal distribution of the data, followed by Dwass-Steel-Critchlow-Fligner (DSCF) pairwise comparisons. Statistical analyses were performed using Jamovi version 2.6.44, with the Rj module for R-based computations. Statistical significance was established at p < 0.05.

For water, sediment, and fish samples, the contribution of each shape, size class, and polymer was calculated based on the total number of microplastics identified within each sample and express as relative frequency (%).

## 3 Results

### 3.1 Microplastics in surface water

A total of 52 microplastics were identified across 10 of the 11 water samples. No microplastics were detected at site W5 (Figure 2A). The highest abundances were observed at W2 (17 MPs L^-1^) and W1 (9 MPs L^-1^). Intermediate values were recorded at W4, W7, and W11 (5 MPs L^-1^), and W8 (4 MPs L^-1^). The lowest concentrations were observed at W3, W6, and W9 (2 MPs L^-1^) and W10 (1 MPs L^-1^).

**FIGURE 2:**
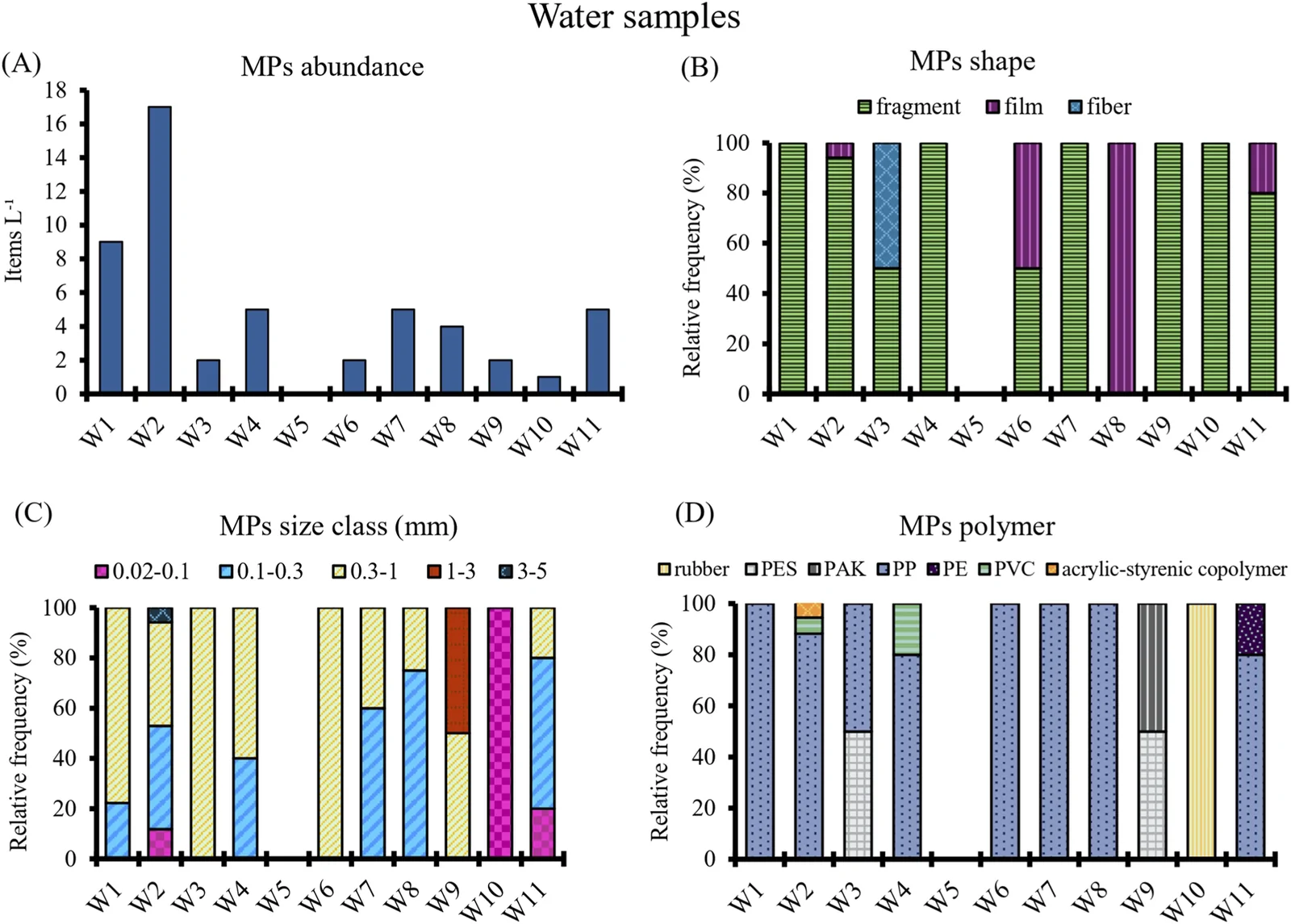
Bar graphs showing the results of microplastic (MPs) analyses in surface water collected from eleven locations in the Gilgit-Baltistan region: **(A)** MPs abundance, reported as the number of particles detected in a single 1-L sample (items L^-1^; n = 1 per site; error bars are not shown because no field replicates were collected), and the relative contributions of **(B)** shapes, **(C)** size classes, and **(D)** polymers to the total MPs identified at each sampling site. PES = polyester, PAK = polyacrylate, PP = polypropylene, PE = polyethylene, PVC = polyvinyl chloride.

Fragments were the dominant shape across most sites, accounting for 80%–100% of microplastics (Figure 2B). Films were present in lower proportions but showed spatial variability, reaching 100% at W8 followed by W6 (50%), W11 (20%) and W2 (6%). Fibers were detected only at W3 (50%; n = 2, one fiber and one fragment).

Regarding particle size, most MPs fell within the 0.1–0.3 mm and 0.3–1 mm size ranges at most sampling sites (Figure 2C). The 0.3–1 mm fraction reached 100% at W3 and W6, with high proportions also recorded at W1 (78%) and W4 (60%). The 0.1–0.3 mm fraction was dominant at W8 (75%), W7 (60%), and W11 (60%). The smallest MPs (0.02–0.1 mm) showed a more limited distribution, with a local peak of 100% at W10, where only one microplastic was detected. This particle (0.023 mm) was also the smallest microplastic of the entire dataset. Lower contributions were recorded at W11 (20%) and W2 (12%). Similarly, larger size fractions were limited in occurrence: the 1–3 mm class was observed only at W9 (50%), while the 3–5 mm class was found at W2 (6%). The maximum recorded size was 3.82 mm for a fiber-shaped MPs.

A total of seven polymer types were identified across samples, including synthetic rubber, polyester (PES), polyacrylate (PAK), polypropylene (PP), polyethylene (PE), polyvinyl chloride (PVC) and acrylic-styrenic copolymer (Figure 2D; Supplementary Figure S1). PP was the predominant polymer at almost all sites, representing 50%–100% of the identified particles with the highest proportion recorded at W1, W6, W7, and W8. Conversely, PP was not detected at W9 and W10, where only two and one microplastic particles were identified, respectively, corresponding to PES and PAK at W9 and rubber at W10.

### 3.2 Microplastics in sediment

Microplastics were detected in 8 out of the 10 sediment samples analyzed, with a total of 34 particles extracted. Concentrations ranged from 55 to 537 MPs kg^-1^ dw. The highest abundance was detected at site S10, followed by S7 (398 MPs kg^-1^ dw) and S5 (383 MPs kg^-1^), while the lowest value was recorded at S8. Sites S1, S2, and S3 exhibited comparable concentrations (99–105 MPs kg^-1^ dw), whereas S9 showed an intermediate abundance (160 MPs kg^-1^ dw) (Figure 3A).

**FIGURE 3:**
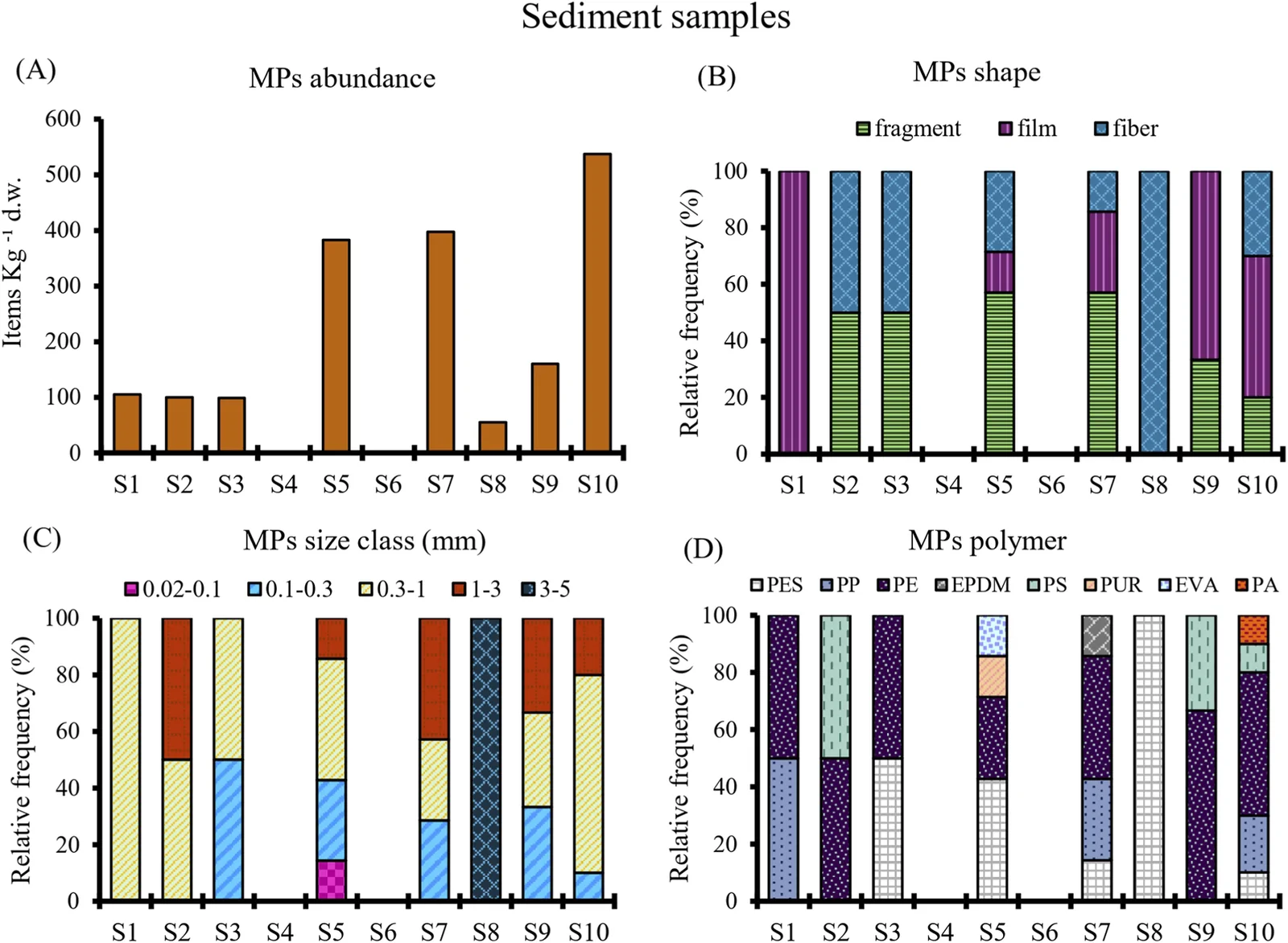
Bar graphs showing the results of microplastic (MPs) analyses in sediment collected from ten locations in the Gilgit-Baltistan region: **(A)** MPs abundance, expressed as the number of particles per kilogram of dry sediment (items kg^-1^ dw; n = 1 per site; error bars are not shown because no field replicates were collected), and the relative contributions of **(B)** shapes, **(C)** size classes, and **(D)** polymers to the total MPs identified at each sampling site. PES = polyester, PP = polypropylene, PE = polyethylene, EPDM = ethylene propylene diene monomer, PS = polystyrene, PUR = polyurethane, EVA = ethylene-vinyl acetate, PA = polyamide.

The relative contribution of different shapes varied among samples, with no single typology consistently dominant across all sites (Figure 3B). Films represented 100% of particles at S1 and accounted for the highest proportion at S9 (67%) and S10 (50%). Fragments predominated at S5 and S7 (57% each), whereas fibers were the only shape detected at S8. Sites S2 and S3 showed equal proportions of fragments and fibers (50% each). All three shape categories were detected at S5, S7, and S10.

The 0.3–1 mm size class was the most abundant, accounting for 100% of microplastics at S1, 70% at S10 and representing the largest proportion at S5 (43%) (Figure 3C). The 1–3 mm size class predominated at S7 (43%) and was also common at S2 (50%) and S9 (33%). Particles in the 3–5 mm size class were detected exclusively at S8 (100%), including the largest microplastic identified, a 3.5 mm fiber. The smallest size class (0.02–0.1 mm) occurred only at S5 (14%) where the smallest particle detected was a 0.09 mm fragment. Sites S3 and S9 exhibited a relatively even distribution of size classes.

Among the eight polymers identified in sediments, PE was the most frequent, occurring in seven of the eight sites where MPs were detected and reaching the highest proportion at S9 (67%) and S10 (50%) (Figure 3D; Supplementary Figure S2). PES predominated at S8 (100%) and S5 (43%), while PP accounted for 50% at S1. Polystyrene (PS) was detected at S2 (50%), S9 (33%), and S10 (10%). Less common polymers, including polyurethane (PUR), ethylene-vinyl acetate (EVA), ethylene propylene diene monomer (EPDM), and polyamide (PA), were recorded only at individual sites. Sites S5 and S10 exhibited the highest polymer diversity, with four and five polymer types identified, respectively, among the eight detected.

### 3.3 Microplastics in fish

Overall, microplastics were detected in 19 out of 31 gastrointestinal tracts analyzed, with a total of 50 particles extracted, including 26 from F1, 12 from F2, 3 from F3, and 9 from F4. The highest frequency of occurrence was recorded at F1 (83.3%), followed by F4 (77.8%), F2 (62.5%) and F3 (25%). However, Fisher’s exact test did not reveal significant differences in the proportion of fish containing MPs among sites (p = 0.10) (Figure 4A). In contrast, the number of ingested microplastics differed significantly among sampling sites (Kruskal-Wallis test, p = 0.009). According to DSCF *post hoc* comparison, fish collected at F1 exhibited significantly higher values (5.2 ± 3.5 MPs individual^-1^) than those collected at F4 (1.3 ± 0.5 MPs individual^-1^). No significant differences were observed among the remaining sites, with fish from F2 and F3 containing 2.4 ± 1.1 and 1.5 ± 0.7 MPs individual^-1^, respectively (Figure 4B).

**FIGURE 4:**
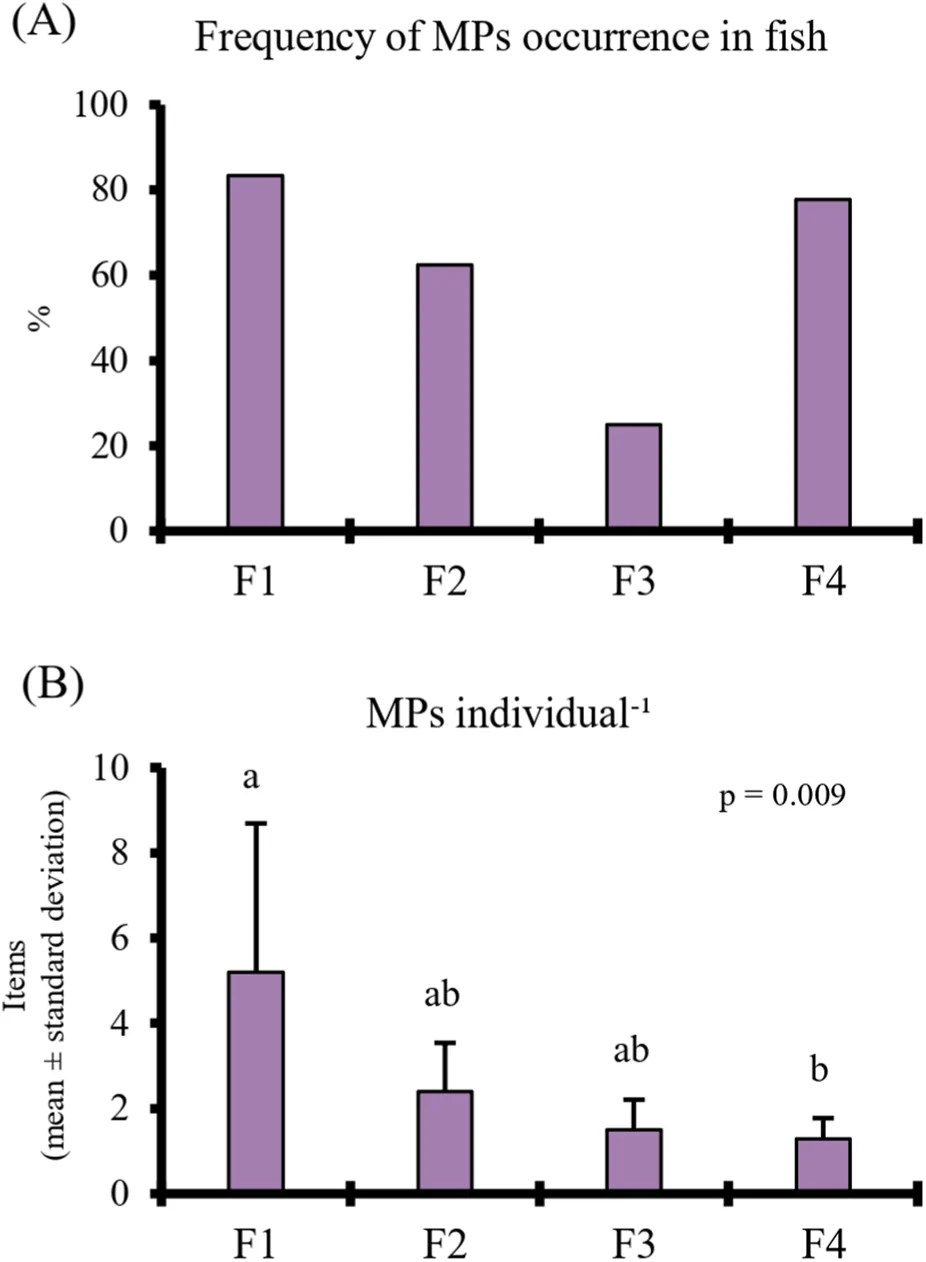
Bar graphs showing the results of microplastic (MPs) analyses in the gastrointestinal tracts of fish collected from four locations in the Gilgit-Baltistan region: **(A)** frequency of occurrence of MPs, expressed as the percentage of individuals containing at least one MP; **(B)** mean number (±SD) of MPs per individual calculated only for fish positive to ingestion. The reported p-value refers to the Kruskal-Wallis test; different letters indicate significant differences among samples (DSCF *post hoc* test).

Fragments and fibers were detected at all sampling sites, whereas films were absent in F3. Fragment-shaped MPs dominated F1 and F3, accounting for 54% and 67% of the total identified particles, respectively. In contrast, films were the predominant shape category in F2 (50%) and F4 (56%). Fibers accounted for 31%, 25%, 33% and 11% of the extracted MPs at F1, F2, F3, F4, respectively (Figure 5A).

**FIGURE 5:**
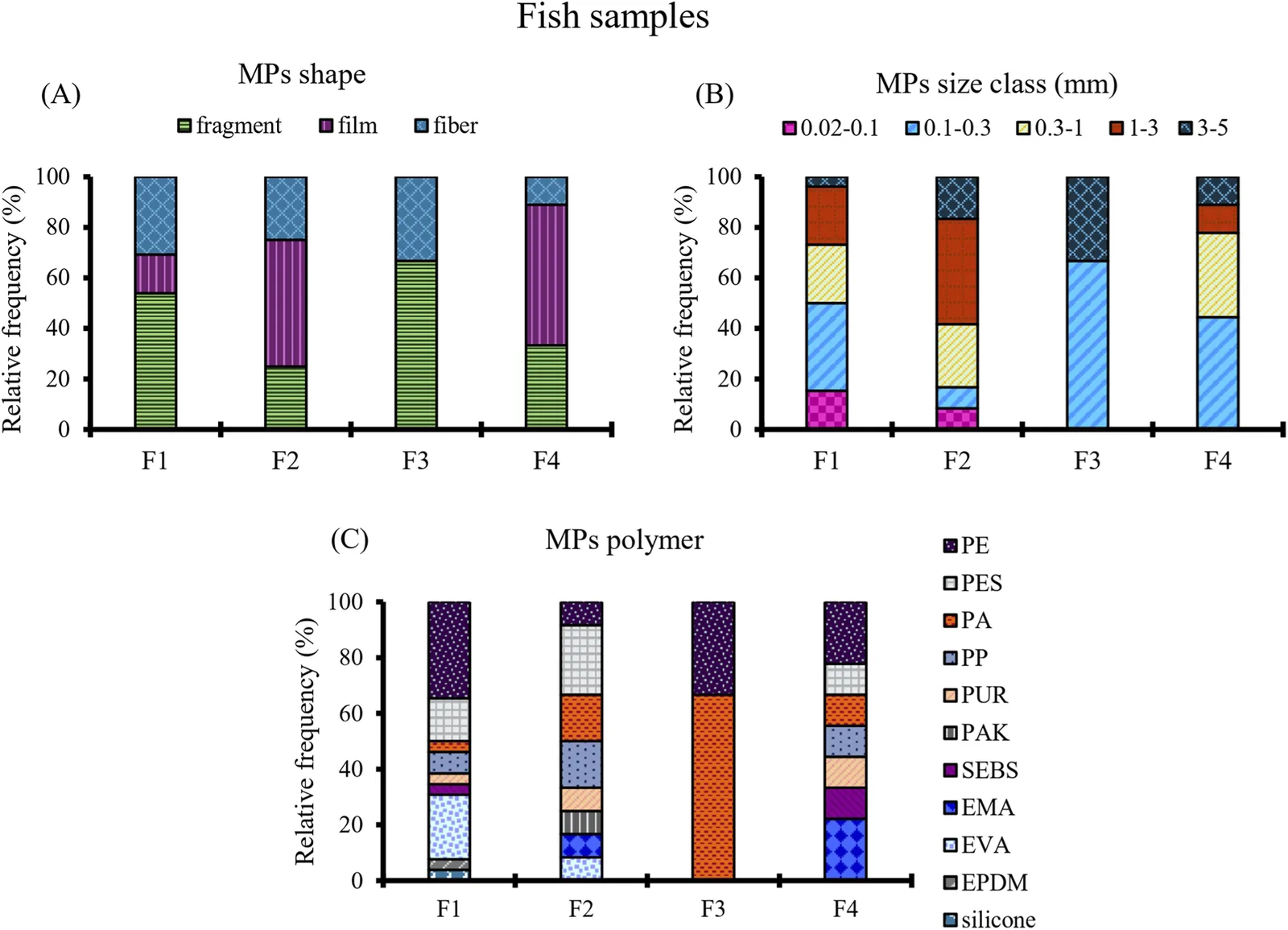
Bar graphs showing the characterization of microplastic (MPs) detected in the gastrointestinal tracts of fish collected from four locations in the Gilgit-Baltistan region: relative contributions of **(A)** shapes, **(B)** size classes, and **(C)** polymers to the total MPs identified at each sampling site. PE = polyethylene, PES = polyester, PA = polyamide, PP = polypropylene, PUR = polyurethane, PAK = polyacrylate, SEBS = styrene-ethylene-butylene-styrene, EMA = ethylene methyl acrylate, EVA = ethylene-vinyl acetate, EPDM = ethylene propylene diene monomer.

MPs in the 0.1–0.3 mm size class were detected at all sampling sites and represented the predominant category in F1 (35%), F3 (67%), and F4 (44%), whereas F2 was dominated by MPs in the 1–3 mm size range (42%). The 0.3–1 mm size class accounted for 23%–33% of the extracted particles in F1, F2, and F4, but was not detected in F3. Similarly, MPs within the smallest size class (0.02–0.1 mm) were recorded only at F1 (15%) and F2 (8%). Largest MPs (3–5 mm size) were found at variable percentage ranging from 4% in F1 to 33% in F3 (Figure 5B). The smallest and largest particles recovered in fish were both recorded at F1 and consisted of a 0.04 mm film and a 4.36 mm fiber, respectively.

Polymer characterization revealed the presence of 11 different polymer types among the extracted MPs: PE, PES, PA, PP, PUR, PAK, styrene-ethylene-butylene-styrene (SEBS), ethylene methyl acrylate (EMA), EVA, EPDM, and silicone (Figure 5C; Supplementary Figure S3). PE and PA were detected at all sampling sites: PE represented 35%, 8%, 33%, and 22% of the particles recovered from F1, F2, F3, and F4, respectively, whereas PA accounted for 4%, 17%, 67%, and 11%. Polymer diversity at F3 was limited to these two polymer types, although only three particles were recovered overall from fish collected at this site. The remaining polymers showed a more restricted distribution. PES represented the most abundant polymer at F2 (25%), whereas EMA was among the dominant polymer at F4 (22%). PAK was detected exclusively at F2, while EPDM and silicone were identified only at F1. EVA occurred only at F1 and F2, whereas SEBS was recorded at F1 and F4.

## 4 Discussion

### 4.1 Microplastic occurrence in water, sediment and fish

Gilgit-Baltistan is a high-mountain region located in northern Pakistan at the confluence of the Karakoram, Himalayan, and Hindu Kush mountain systems, hosting extensive glacier-fed rivers and freshwater ecosystems that play a critical role in sustaining downstream water resources within the Indus River basin. Our findings revealed the ubiquitous occurrence of MPs across the sampled sites during the study period. Moreover, the adopted multi-matrix approach demonstrated that relying on a single environmental compartment may provide an incomplete picture of microplastics contamination, as each matrix reflects different environmental processes and captures complementary temporal and spatial dimensions of contamination (Campanale et al., 2020). For example, MPs were not detected in water collected from a tributary of Shigar River (W5) while they were found in sediments (S5); the opposite was observed at Braldu and Shigar rivers (W4-S4 and W6-S6, respectively). These differences likely reflect the distinct environmental dynamics represented by each compartment: water samples primarily capture recent inputs and ongoing transport processes, whereas sediments represent longer-term sink integrating historical deposition and accumulation (Bhaduri et al., 2025; Martin et al., 2023; Lahon and Handique, 2023). Fish, in turn, reflects environmental exposure and potential trophic transfer. Findings from Satpara Lake further highlight the value of the integrated approach. At this site water sample (W10) showed the lowest MPs abundance among the investigated sites, consistent with the lowest frequency of MPs occurrence observed in wild individuals of *S. skarduensis* (F3). In contrast, sediment (S10) exhibited the highest concentrations recorded in the study area, suggesting that elevated levels of MPs in abiotic compartments does not necessarily correspond to higher rate of particles ingestion, even in benthopelagic species, as biological uptake may also be influenced by feeding strategy, habitat preference, and particles bioavailability (Gholizadeh and Bagheri, 2026).

Beyond differences in occurrence, microplastic characteristics also showed clear matrix-dependent variability. MPs in the shape of fragments and in the size below 1 mm dominated in water samples, suggesting a substantial contribution from the weathering and breakdown of larger plastic items and their progressive fragmentation into smaller particles. Moreover, the predominance of PP is consistent with its widespread use in packaging, disposable products, piping application, while its persistence in surface waters is likely favored by the buoyancy-driven transport of this low-density polymer (Bouida et al., 2026). In contrast, both sediments and fish displayed a more balanced contribution of fragments, films, and fibers, a broader size spectrum including larger MPs (>1 mm), and a more heterogeneous polymer composition compared to surface waters. In sediments, these patterns are consistent with their role as depositional environments, reflecting the progressive accumulation and retention of particles from multiple sources over time (Lahon and Handique, 2023). Films may originate from the degradation of plastic bags, packaging materials, and other thin plastic items, whereas fibers are commonly associated with synthetic textiles and other fibrous materials and may enter freshwater systems through wastewater discharge, surface runoff, or atmospheric deposition (Bexeitova et al., 2024; Zhao et al., 2024). The relative enrichment of larger microplastics is consistent with size-dependent transport processes, as sedimentation rates generally increase with particle size (Horton et al., 2017). In addition, larger particles are more prone to biofilm formation and aggregation with suspended matter, which enhance their effective density and promotes settling (Li et al., 2023; Bilal et al., 2023a; Campanale et al., 2020). Through these same processes, low-density polymers may settle over time and accumulate alongside denser polymers (Bexeitova et al., 2024), explaining the co-occurrence of polymers with contrasting intrinsic densities in sediments. Accordingly, PE was the most widespread identified polymer, although several other low-density (PP, EVA, EPDM) and high-density (PES, PS, PUR, PA) polymers were also detected, indicating that MPs composition in sediment samples may reflect the combined influence of both particle-specific properties and environmental processes (Pittura et al., 2023). In fish, however, the typologies of ingested microplastics are influenced not only by environmental availability but also by specific processes acting before and after ingestion, such as prey-mediated transfer, selective ingestion, and differential retention within the gastrointestinal tract; also, the gut content reflects only the instantaneous diet (Collard et al., 2019). This complexity is particularly reflected in the polymer composition, as fish samples exhibited the highest polymer diversity among the investigated matrices, with 11 polymer types identified compared with seven in surface waters and eight in sediments.

The occurrence of microplastics across all investigated compartments provides a valuable baseline for a previously understudied high-altitude region. To contextualize these findings, observed abundances were compared with those reported in similar freshwater systems, although direct comparisons among studies should be interpreted cautiously because methodological differences can substantially influence reported MPs concentrations.

For water samples the range of 1–17 particles L^-1^ observed in the present study is comparable to values reported for alpine lakes in Gilgit-Baltistan (2–17 MPs L^-1^, Mehboob et al., 2024), Lake Sassolo in Switzerland (2.6–4.4 MPs L^-1^, Negrete Velasco et al., 2020), and Spanish lakes (0.3–21.3 MPs L^-1^, Godoy et al., 2022). Lower concentrations have been reported in water of freshwater systems across the Tibetan Plateau-Himalayan region, typically <3 MPs L^-1^ (Jatav et al., 2025; Wang Y. et al., 2023), consistent with the lower end of the range observed in the present study. In contrast, substantially higher concentrations have been reported in the Swat River in Pakistan (92–192 MPs L^-1^; Khan et al., 2022) and the Beas River in western Himalayas (46–222 MPs L^-1^; Bhaduri et al., 2025), exceeding the upper concentrations observed in the present study by approximately one order of magnitude.

The MPs concentrations detected in sediment samples (55–537 MPs kg^-1^ dw) partially overlap those reported for lakes in Gilgit-Baltistan (12.1 ± 7 to 152.6 ± 104.6 MPs kg^-1^ dw, Mehboob et al., 2024). Comparable values have also been reported across several freshwater systems of the Tibetan Plateau-Himalayan region, including the Upper Brahmaputra basin (80–90 MPs kg^-1^dw), the Yangshiping-Lhasa catchment (50–195 MPs kg^-1^ dw), the Qilian Mountains (20–160 MPs kg^-1^ dw), the Koshi River (15–120 MPs kg^-1^ dw), and the Lesser Himalayas (15–632 MPs kg^-1^ dw) (Wang T. et al., 2023). In contrast, higher concentrations have been reported in major river systems of the Indian Himalaya, including the Indus River and downstream of Brahmaputra River (698–1987 and 650–3,494 MPs kg^-1^ dw, respectively; Tsering et al., 2021), as well as in Anchar Lake in the Kashmir Valley, Northwest Himalaya (233–1,533 MPs kg^-1^ dw; Neelavannan et al., 2022).

Microplastic ingestion in fish ranged from 1.3 ± 0.5 to 5.2 ± 3.5 MPs individual^-1^ across the sampled species (*O. mykiss*, *S. trutta*, and *S. skarduensis*). Comparable levels have been reported in Schizothorax spp. from Tibetan Plateau river systems, with ingestion levels of approximately 3.9–7.6 MPs individual^-1^ (Wang et al., 2026). Similar values have also been observed in cold-water salmonids, including rainbow trout (*O. mykiss*) and brown trout (*S. trutta*), from the Mendoza River (Central Andes, Argentina; ∼2–3 MPs individual^-1^; Rios et al., 2022) and rainbow trout from the Chehel Chai River (Iran; 1.57 ± 0.29 MPs individual^-1^; Gholizadeh and Bagheri, 2026). Higher values have been reported for *Schizothorax plagiostomus* from the Swat River system with 17.08 ± 8.27 MPs individual^-1^ (Bilal et al., 2023a), although these may represent an overestimation due to the absence of polymer characterization.

### 4.2 Spatial patterns and potential drivers of microplastic distribution across riverine and lake environments

By encompassing remote headwaters, main river channels, tributaries, lentic systems, and human-influenced areas, the present study revealed spatial variability in microplastics contamination across freshwater environments in the study area, elucidating the main processes governing MPs distribution. Consistently, MPs concentrations did not exhibit a simple downstream increasing trend along the Braldu-Shigar-Indus river continuum, a pattern previously reported in several river systems and typically linked to increasing anthropogenic pressure along the fluvial gradients (Alessanta et al., 2026; Sulistyowati et al., 2022; Tsering et al., 2021; Singh et al., 2021; Eo et al., 2019; Luo et al., 2019). Instead, microplastic occurrence appeared to be strongly influenced by local inputs and hydrological conditions, resulting in a patchy distribution across the catchments. However, given that only one sample was collected per site for each abiotic matrix, the observed spatial patterns cannot account for within-site variability in microplastic concentrations.

The highest microplastics abundances in surface water were recorded at the Braldu River tributary in Korphe (W2) followed by the small stream in Askole (W1). Both locations are situated in the upper Braldu Valley, where small permanent settlements coexist with intense summer tourism driven by trekking within the Central Karakoram National Park and mountaineering to the surrounding eight-thousanders. This seasonal influx of visitors may increase generation of plastic waste through domestic activities, camping, and logistical support for expeditions (Napper et al., 2020). Tourism has been recognized as one of the major sources of microplastic pollution in mountain environments, as plastic items such as food and beverage packaging, disposable materials, and mountaineering equipment may be intentionally or unintentionally abandoned along trekking routes and expedition camps (Chauhan and Banerjee, 2025). Moreover, in remote mountain settlements such as Askole and Korphe, these additional inputs may overwhelm local waste management capacities, which are constrained by difficult topography, inadequate infrastructure, and limited waste collection and disposal services (Ali, 2023). This potential contribution of tourism is consistent with the findings of Saqib et al. (2019), who reported that local communities in Gilgit-Baltistan perceive tourism as the primary driver of plastic waste accumulation in the surrounding environment.

In contrast, microplastic levels in the main Braldu River (W3) were markedly lower, suggesting that greater streamflow, dilution capacity, and turbulence-driven mixing may influence MPs detectability (Guo et al., 2024). Although road traffic at the Korphe Bridge, situated immediately upstream of the sampling site, may represent a potential source of microplastics, including tire wear particles (Gonzalez-Saldias et al., 2024), no clear contribution was detected at this location. This reinforces the transient nature of surface water samples, which reflect recent inputs and active transport processes rather than long-term accumulation, as previously highlighted, particularly in larger river channels. A contrasting pattern was observed in the Shigar River, where microplastics were absent in the tributary site (W5) but present at low concentrations in the main river channel (W6; 2 MPs L^-1^). The lack of MPs in W5 may indicate either very limited local sources or temporary retention of particles upstream, while their presence in W6 suggests that microplastics are delivered from different sub-catchments in an irregular and episodic manner. Therefore, microplastics occurrence in the investigated Shigar River likely reflects irregular inputs and a strong dependence on hydrological connectivity between tributaries and the main channel. In the Skardu area, the Indus River sampling site (W7) is located immediately downstream of the tributary confluence (W8), which likely explains the similar microplastic concentrations (5 and 4 MPs L^-1^, respectively), reflecting local connectivity and rapid integration of tributary inputs into the main channel. Despite the higher population density and more intense human activity in the Skardu area, MPs concentration remained relatively low, suggesting that greater dilution associated with the larger discharge of the Indus River, together with hydrological transport processes, may play a stronger role than direct anthropogenic inputs in controlling observed patterns.

Overall, the spatial pattern observed in water samples can be interpreted within the geomorphic context of the Braldu-Shigar-Indus system described by Seong et al. (2009), which is characterized by glacier-fed hydrology and strong spatial variability in channel morphology. Alternating zones of incision and sediment storage create a heterogeneous fluvial network, where particulate transport, including microplastics, is not spatially uniform but controlled by transfer, storage, and re-mobilization dynamics. Within this framework, results on sediment samples further indicate a decoupling between transport and accumulation, with contrasting patterns between water and sediments. Higher concentrations in water (W1-W2) correspond to lower sediment loads (S1-S3), while W5 shows the opposite pattern. These findings emphasize the importance of distinguishing between transport and storage domains when assessing microplastic distribution in mountain river systems. The observed shape composition may further contribute to this spatial variability because MP morphology can influence transport and retention across the different environmental compartments (Guo et al., 2024). Fragments may remain relatively mobile in the water column, particularly under high-flow conditions, but can be transferred to sediments as flow velocity decreases or within local sediment-storage zones (Gao et al., 2025). Films may also be transported downstream but, because of their low mass and large surface area, can be readily redistributed by turbulence and surface-water movement while subsequently being retained in depositional areas where reduced flow velocities may promote their accumulation in sediments (Croiset et al., 2024; van Emmerik and Schwarz, 2020). Fibers are similarly highly transportable: their elongated morphology and relatively low settling velocities may favor prolonged transport in the water column, although biofouling and aggregation with organic matter or mineral particles can promote their deposition in sediments (Nguyen et al., 2022; Leiser et al., 2020). This framework may help explain why the relative contribution of different MPs shapes varies between water and sediment compartments and among sites, as particles generated upstream can be transported, temporarily stored, and subsequently remobilized depending on local flow conditions and sediment-storage dynamics.

The Satpara Lake system further supports the contrasting behavior observed between water and sediments throughout the study area. Surface water collected from the lake (W9) and the Satpara spring (W10) showed very few MPs, whereas sediments, especially near the Satpara Stream inflow (S10), showed the highest MP concentrations recorded in the entire study. Despite being a popular tourist destination, Satpara Lake is not directly affected by the urban area of Skardu and is fed by streams draining the Deosai Plateau rather than the Braldu-Shigar-Indus catchment, which may carry lower microplastic loads. The transition from fluvial to lacustrine conditions, combined with the generally more stable hydrodynamic conditions of lentic environments, lower flow velocities, and biofouling processes, promotes particle settling and long-term retention, reflecting the well-established role of lentic environments as effective sinks for microplastics (Wang T. et al., 2023). Interestingly, the Satpara Dam outlet (W11) showed higher microplastic concentrations than those measured in the lake itself. Although no sediment sample was collected at this site, this pattern suggests that dam-related hydrodynamics may influence local microplastic distribution. Water release and flow regulation can modify particle transport through resuspension, retention, and selective downstream export, with dams acting as local hotspots for microplastics redistribution within freshwater systems (Gao et al., 2025). Given that the dam outlet feeds the Satpara Stream, which subsequently joins the Indus River, these processes may also influence downstream microplastics transfer from the lake to the fluvial network.

### 4.3 Microplastics ingestion in wild and farmed fish

The presence of microplastics in the gastrointestinal tracts indicates that anthropogenic particles are available for ingestion by freshwater fish in the investigated area. Nevertheless, individuals collected from breeding sites (F1 and F4) showed a higher frequency of occurrence than wild-caught individuals (F3), a pattern consistent with previous reports from the Tibetan Plateau (Wang et al., 2026) and the River Ganga (India) (Kumari et al., 2023), suggesting that farming conditions may increase exposure to microplastics. However, this comparison should be interpreted cautiously as the wild-fish group comprised a limited number of individuals collected from a single sampling site, with only three microplastic particles recovered. Therefore, the lower prevalence observed in wild fish at F3 should not be interpreted as representative of wild fish populations across the study area. Rather, these findings provide a site-specific indication of microplastics ingestion.

Microplastics in aquaculture systems originate from multiple pathways, including land-based inputs such as wastewater discharge, agricultural runoff, and riverine transport, which act as major conduits of plastic debris from inland areas (Wu et al., 2023). Additional local sources include tourism-related littering and the degradation of farming infrastructure (e.g., nets, ropes, pipes, cages and floating devices), as weathering and mechanical wear of these materials can generate secondary microplastics within aquaculture facilities (Wu et al., 2022). Plastic containers, bins, and packaging materials used for fish feed storage, transportation, and handling may contribute to local microplastic inputs through their degradation and improper disposal (Nabi et al., 2024). In addition, aquaculture feeds may represent a direct pathway of microplastic exposure, as contamination can occur through feed ingredients and during processing and handling. The detection of microplastics in both feed ingredients, such as fishmeal, and commercial aquaculture feeds supports feed as an additional exposure pathway for farmed fish (Jeyasanta et al., 2024; Su et al., 2024; Walkinshaw et al., 2022). Also, atmospheric deposition emerges as a significant pathway for microplastic input, particularly in remote environments (Le et al., 2024). Farmed fish remain confined within the same production facilities throughout their life cycle, which can lead to continuous exposure to local contamination sources, whereas wild fish exploit more heterogeneous habitats and feeding resources (Wang et al., 2026; Miao et al., 2023). Fish farming in Gilgit-Baltistan is largely based on cold-water rainbow trout (*O. mykiss*) and brown trout (*S. trutta*), supported by the region’s glacier-fed rivers, springs, and lakes (Laghari, 2018; Edwards, 1991). Both farming locations investigated in the present study rear these salmonid species but operate within different settings and hydrological contexts. Site F1 represents a well-established spring-fed trout hatchery characterized by a regulated water supply and infrastructure designed to support intensive trout production and juvenile distribution for restocking programs (FAO, 1992). In contrast, site F4 is in the upper sector of Satpara Lake, near the inflow of the glacier-fed Satpara Stream, representing a more open aquaculture setting with greater hydrological connectivity than the hatchery system. These contrasting farming environments may partly explain patterns observed in MPs occurrence. Although the frequency of ingestion was high in fish from both sites, individuals from the hatchery exhibited significantly higher number of ingested items compared with those from the lake-associated system. This suggests that, while exposure to microplastics is widespread across farming environments, the load of ingestion differs between systems. The more confined hatchery conditions may promote sustained exposure to microplastics from local contamination sources, whereas the continuous renewal of glacier-fed water in the lake environment may favor particle dispersion, resulting in lower local concentrations and greater spatial heterogeneity in exposure, resulting in reduced microplastics burden.

Fish originating from aquaculture systems have been reported to exhibit higher frequencies of microplastic ingestion than both wild-caught and market-sourced fish (Wootton et al., 2021). In our study, fish purchased from the local market (F2) showed an intermediate frequency of microplastic occurrence between farmed and wild individuals. Although the provenance of individual specimens could not be verified, the species composition of the market samples (including farmed salmonids, *O. mykiss* and *S. trutta*, as well as the native cyprinid *S. skarduensis*) suggests contributions from both aquaculture and wild fisheries. Consequently, F2 provides an integrated picture of MPs exposure experienced by fish entering the local food chain rather than reflecting a single environmental setting.

The patterns observed in microplastic characteristics further support differences in exposure among sampling sites. Fragments and fibers occurred across all groups, consistent with secondary microplastics dominating freshwater environments (Li et al., 2023), whereas films were absent only from the three MPs recovered from wild fish at F3, potentially reflecting differences in local environmental conditions and transport dynamics. The distribution of these shapes may also provide insights into the contribution of the potential sources described above. Fragments may result from the weathering and mechanical degradation of larger plastic items, potentially including aquaculture infrastructure containers and packaging used for feed storage and handling. The occurrence of films may reflect the degradation of thin plastic materials, such as packaging and plastic bags associated with food handling, tourism-related activities and improper waste disposal. Fibers, in turn, may be associated with the degradation and shedding of synthetic ropes and nets used in aquaculture and fishing activities, with atmospheric deposition providing an additional pathway for textile-derived fibers to reach freshwater environments. Thus, the observed mixture of fragments, films, and fibers may reflect the combined contribution of aquaculture-related materials and local diffuse inputs, whose relative contribution may vary with human activities and hydrological connectivity. The smallest size class (0.02–0.1 mm) and the full range of size classes were detected only in F1 and F2. In F1, this pattern may reflect continuous exposure across size classes under confined rearing conditions, whereas in F2 it likely reflects the mixed provenance of market fish and the resulting heterogeneous exposure sources.

Among polymers, PE and PA were detected across all groups, indicating widespread occurrence in the sampled fish. Notably, these were the only polymers identified for the three MPs recovered from wild fish at F3. This is consistent with the extensive use of polyethylene in packaging and aquaculture-related plastics, and of polyamide in synthetic textiles and fishing gear, which are common sources of microplastics and widely reported in freshwater environments (Wu et al., 2023; Neelavannan et al., 2022; Horton et al., 2017). Farmed fish from F1 and F4 showed largely similar polymer profiles, sharing six polymer types (PE, PES, PA, PP, PUR, and SEBS), suggesting broadly comparable exposure to a common background of polymer contamination. The occurrence of a few site-specific polymers (F1: EVA, EPDM, and silicone; F4: EMA) may reflects local inputs related to differences in environmental conditions and management practices, although specific sources cannot be identified from polymer composition alone. Market-sourced fish (F2) displayed a composite polymer profile, including the most widespread polymers, polymers shared with farmed fish (i.e., EVA and EMA), and the exclusive occurrence of PAK, reflecting heterogeneous exposure pathways linked to their mixed provenance. Given the low number of particles recovered from wild fish at F3, the observed patterns in MP shape, size, and polymer composition warrant cautious interpretation.

## 5 Conclusion

This study provides the first multi-compartment assessment of microplastics contamination across glacier-fed freshwater ecosystems in the Gilgit-Baltistan region (northern Pakistan), a high-altitude area that remains largely underrepresented in global assessments. MPs were detected across nearly all sites and compartments, confirming their pervasive occurrence even in remote mountain environments.

Spatial variability was driven by the interplay between hydro-geomorphological processes and local anthropogenic pressures. Local inputs associated with settlements and tourism contributed to elevated concentrations in tributaries, while glacier-fed rivers mainly acted as transport pathways. In contrast, low-energy environments such as Satpara Lake functioned as sinks, promoting microplastics retention and sediment accumulation. No consistent downstream increase was observed, highlighting the dominance of local inputs and hydrological discontinuities.

Distinct matrix-dependent patterns emerged: surface waters were dominated by fragments consistent with secondary microplastics and short-term inputs; sediments showed broader size and polymer diversity, and fish exhibited the highest polymer richness, confirming the entry of MPs into the aquatic food web and multiple exposure pathways.

Microplastics were detected in both wild and farmed fish, with occurrence appearing higher in aquaculture sites, while market-sourced fish reflected mixed exposure sources. The complementary patterns among farmed, wild, and market-sourced fish highlight the importance of integrating multiple fish sources to obtain a more comprehensive picture of exposure pathways within the local aquatic ecosystem.

Despite limitations related to the absence of replicates for abiotic matrices, single-season sampling, and the restricted sampling of wild fish, this study provides a baseline for a data-scarce region, specifically the Braldu-Shigar-Skardu freshwater system. The low number of positive wild fish samples warrants caution when drawing conclusions about microplastic distribution in wild populations. Furthermore, as all samples across the different compartments were collected in June 2024, the findings represent a snapshot of microplastic contamination during this period and may not fully capture seasonal variability. Changes in hydrology, precipitation, tourism intensity, and meltwater dynamics throughout the year may influence microplastics transport, deposition, and ingestion, potentially affecting the observed contamination patterns. Therefore, multi-season sampling, together with replication of abiotic matrices, broader spatial coverage, and increased sample sizes for wild fish, is needed to better assess temporal and spatial variability and improve the representativeness of future assessments.

Nevertheless, the results support evidence-based decision-making and targeted mitigation strategies, including improved waste management and measures to reduce plastic inputs in settlements and other areas affected by local anthropogenic pressures. Community awareness initiatives promoting appropriate waste disposal and reducing plastic use, together with good practices in tourism and aquaculture aimed at minimizing littering, may further contribute to reducing microplastic contamination in the aquatic system. These measures, together with continued microplastics monitoring, could strengthen the scientific basis for the responsible management of water and fishery resources and support long-term assessment of high-altitude freshwater ecosystems that may act as sentinels of environmental change.

## Data Availability

The original contributions presented in the study are included in the article/Supplementary Material, further inquiries can be directed to the corresponding authors.
