# Supplementary material for: Microplastics in freshwater environments across the high altitude region of Gilgit-Baltistan, Pakistan
Source: Front Toxicol. 2026 Sep 22;8:1947679. doi: 10.3389/ftox.2026.1947679 (PMC13639269; doi:10.3389/ftox.2026.1947679)
Supplement: Supplementary file 1 [file Image1.pdf]

Supplementary Material

(A)

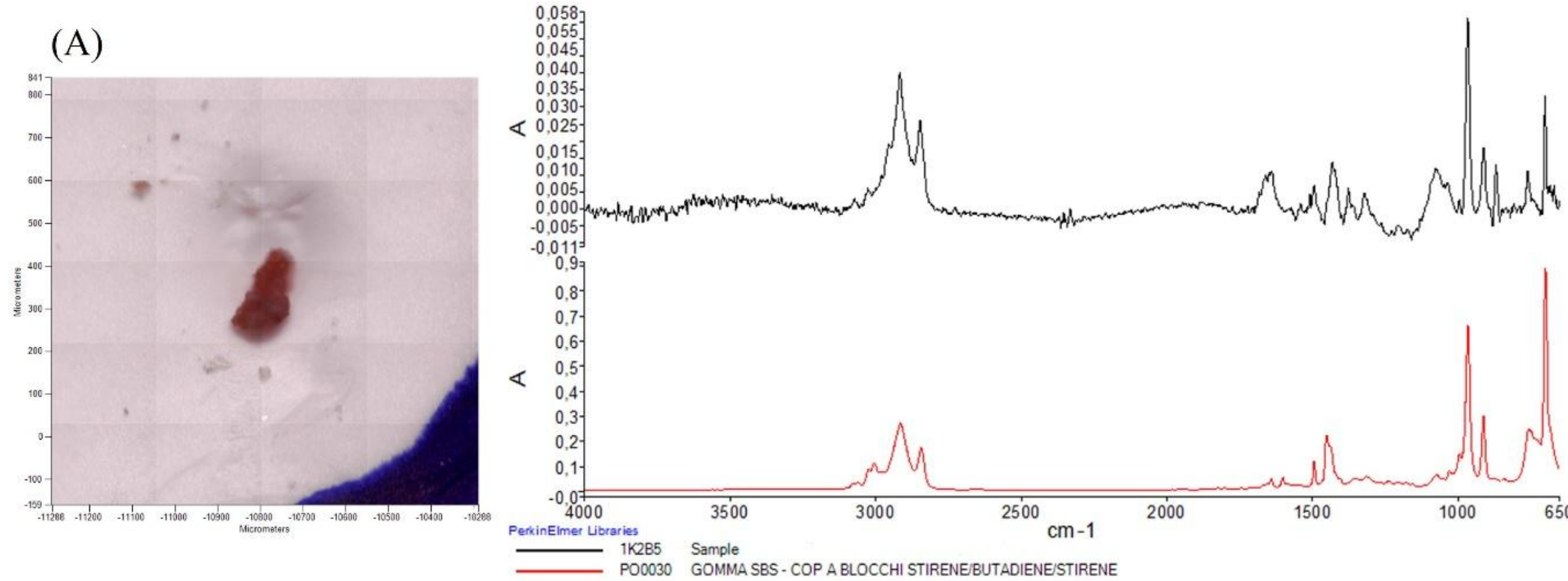

Source Spectra				
**Sample Name**	**Search Score**	**Search Best Hit**	**Search Best Hit Description**	**Search Library**
1K2B5	0,804599	PO0030	GOMMA SBS - COP A BLOCCHI STIRENE/BUTADIENE/STIRENE	PERKIN~1

(B)

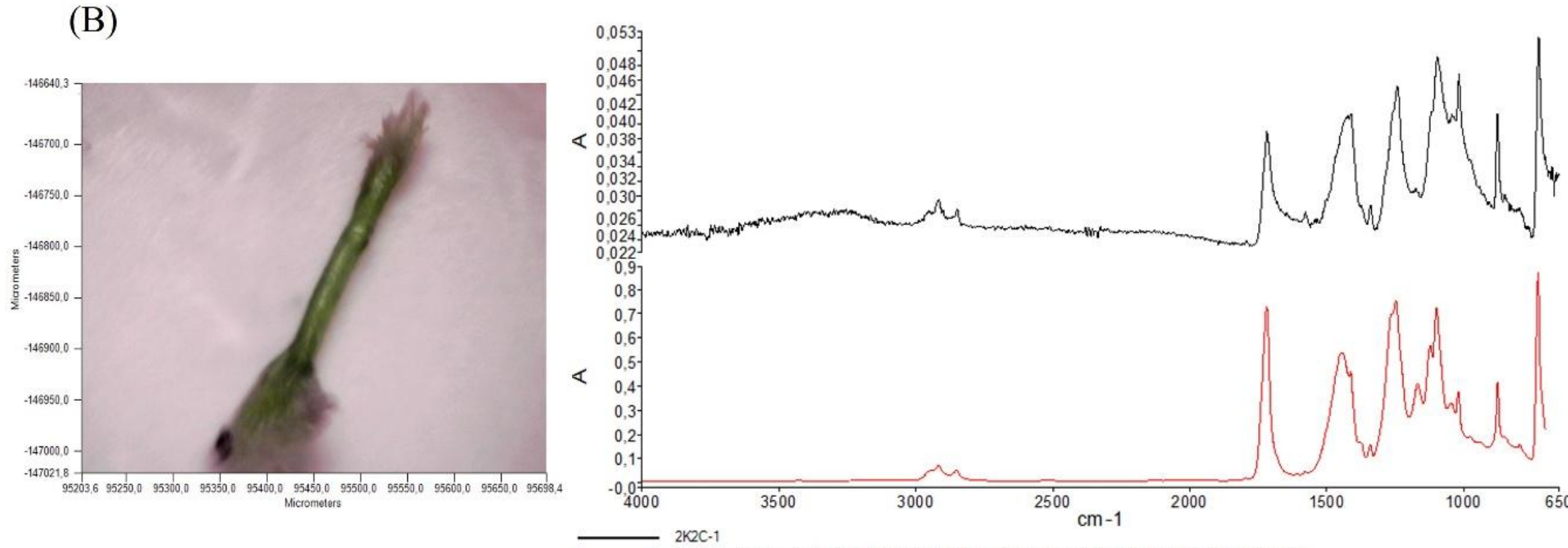

Source Spectra				
**Sample Name**	**Search Score**	**Search Best Hit**	**Search Best Hit Description**	**Search Library**
2K2C-1	0,865743	PO0111	VITEL - POLIESTERE TERMOPLASTICO, LINEARE,ALTO PESO MOLECOLARE (+CACO3)	POLIMERI

(C)

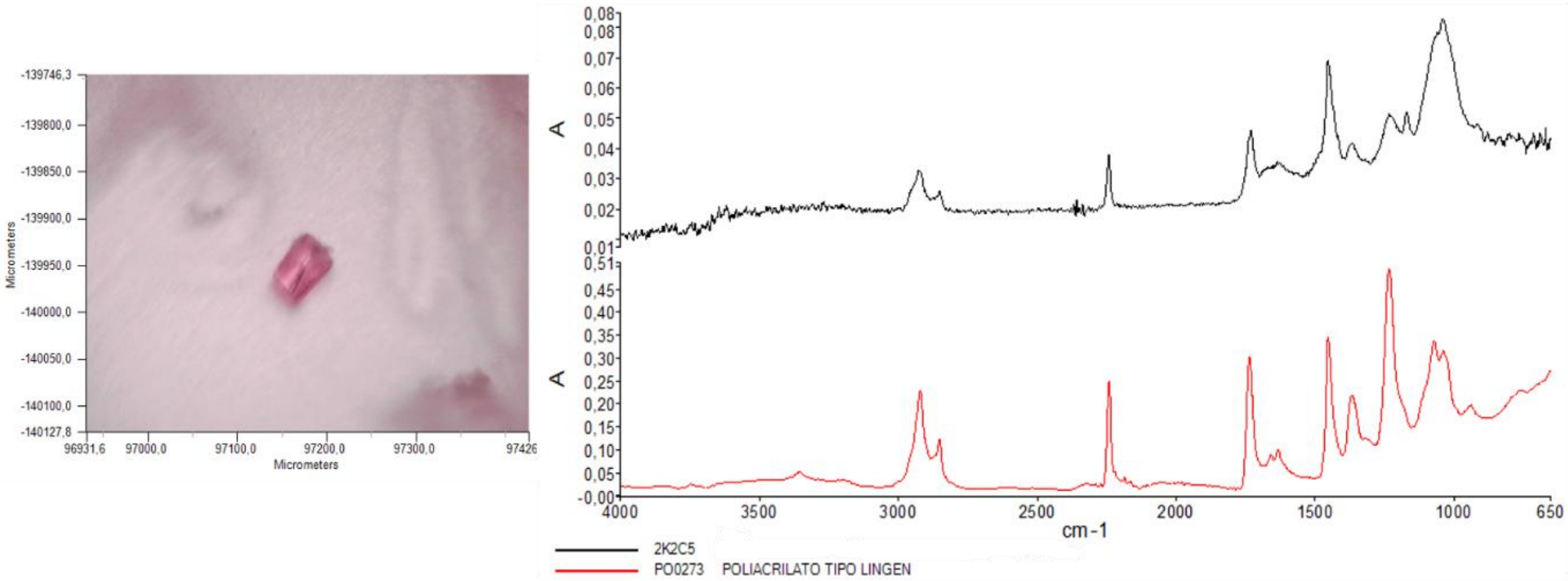

Source Spectra				
Sample Name	Search Score	Search Best Hit	Search Best Hit Description	Search Library
2K2C5	0,798813	PO0273	POLIACRILATO TIPO LINGEN	PERKIN~1

(D)

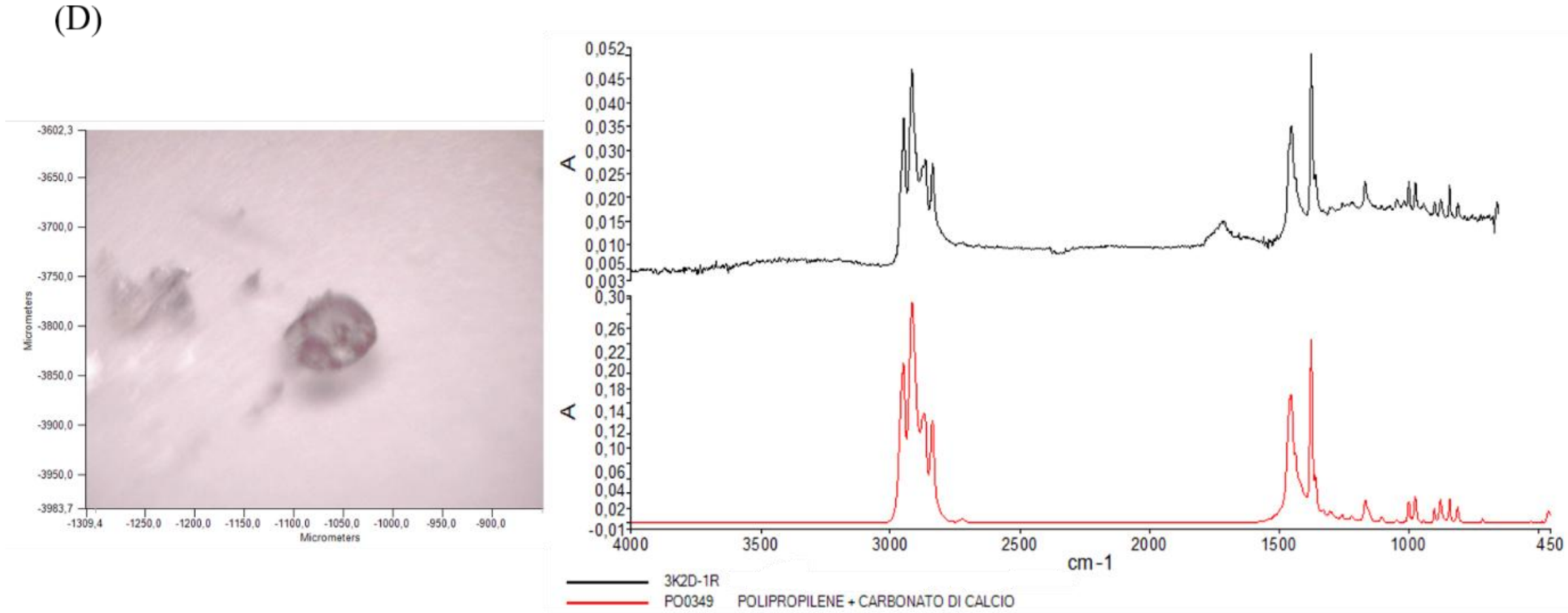

Source Spectra				
Sample Name	Search Score	Search Best Hit	Search Best Hit Description	Search Library
6K2 H2O.13 PP	0,954509	PO0348	PP RACO (RANDOM COPOLIMER) - UATR DI-KRS5	POLIMERI

(E)

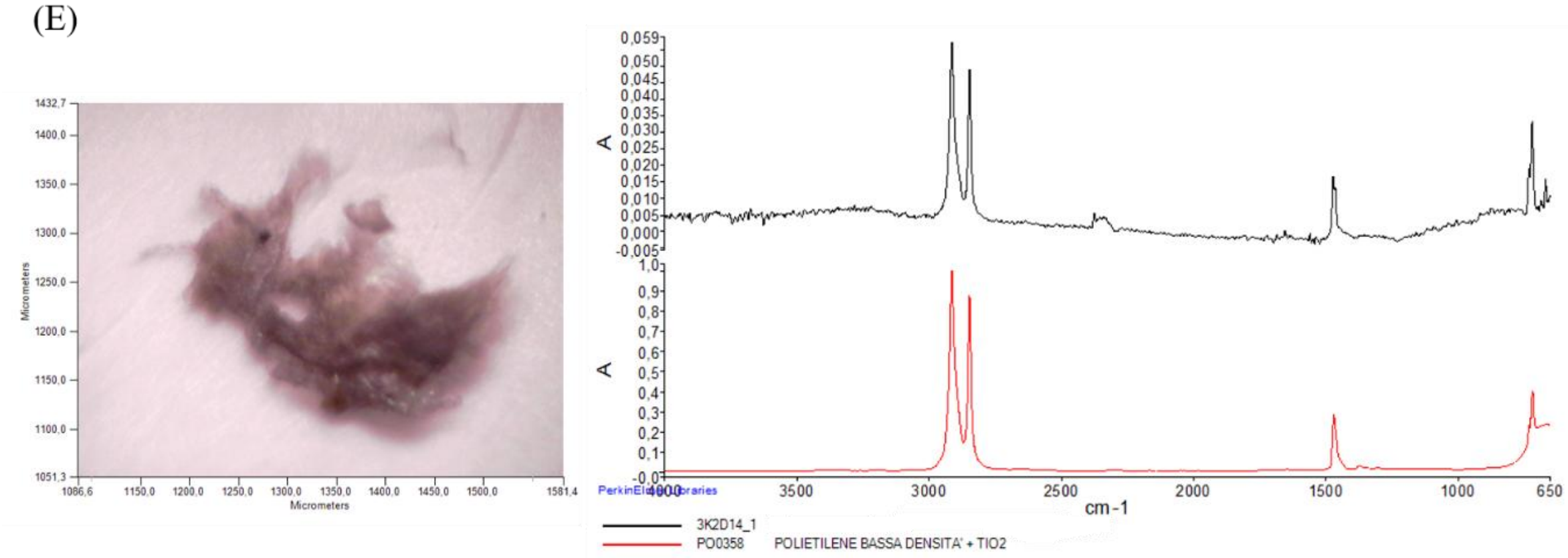

Source Spectra				
**Sample Name**	**Search Score**	**Search Best Hit**	**Search Best Hit Description**	**Search Library**
3K2D14_1	0,967909	PO0358	POLIETILENE BASSA DENSITA' + TIO2	PERKIN~1

(F)

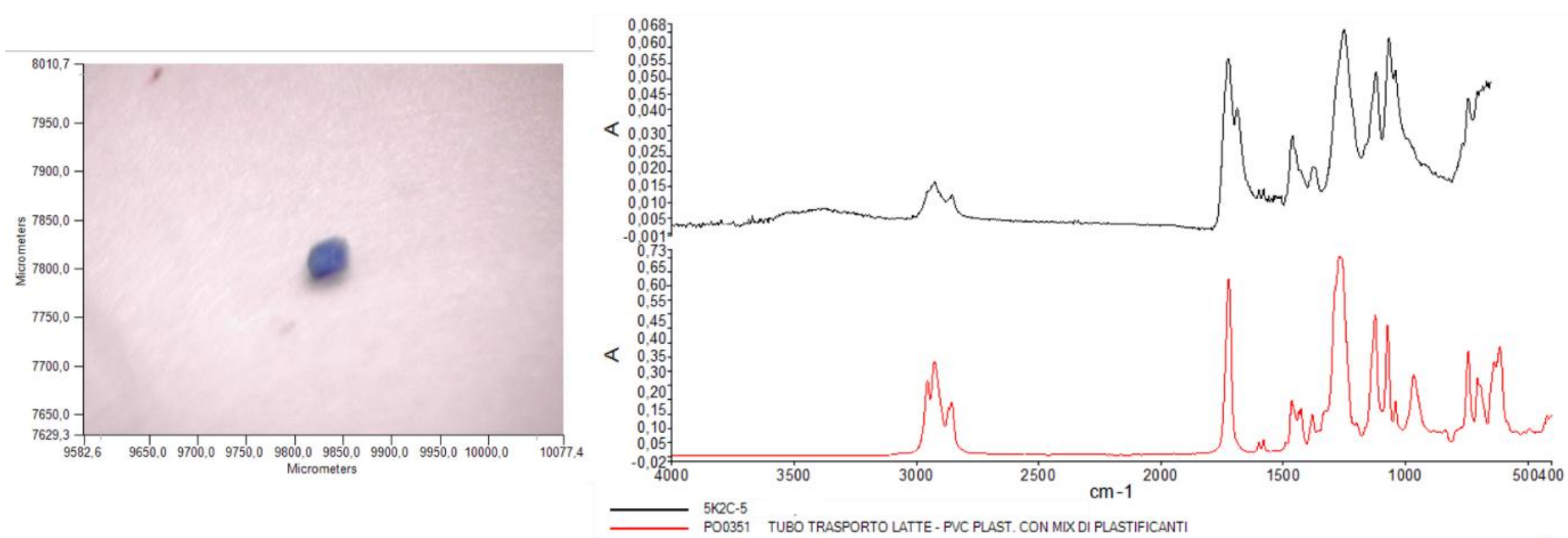

Source Spectra				
**Sample Name**	**Search Score**	**Search Best Hit**	**Search Best Hit Description**	**Search Library**
5K2C-5	0,768427	PO0351	TUBO TRASPORTO LATTE - PVC PLAST. CON MIX DI PLASTIFICANTI	PERKIN~1

(G)

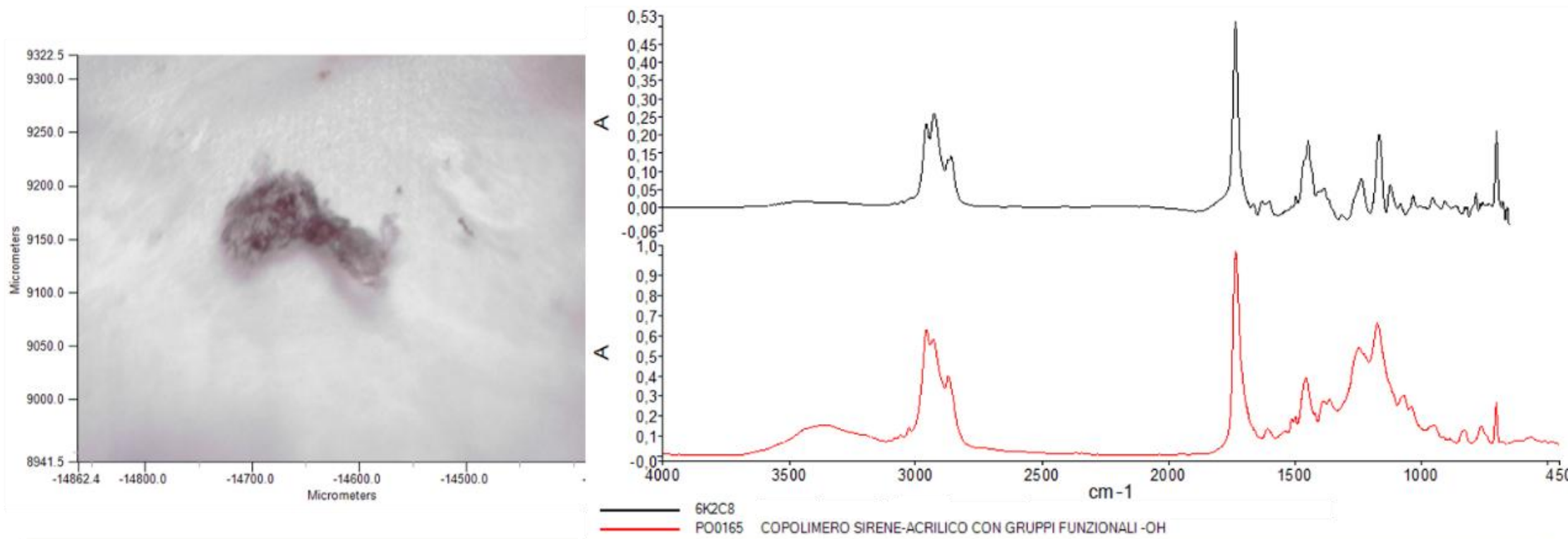

Source Spectra				
Sample Name	Search Score	Search Best Hit	Search Best Hit Description	Search Library
6K2C8	0,869154	PO0165	COPOLIMERO SIRENE-ACRILICO CON GRUPPI FUNZIONALI -OH	POLIMERI

Supplementary Figure 1. Representative μ-FTIR spectra of microplastic particles identified in water samples. One representative particle is shown for each of the seven polymer types identified, along with its corresponding μ-FTIR spectrum (black) and the reference spectrum from the spectral library (red). The polymer identification and library match score are reported for each particle. (A) rubber, (B) polyester, (C) polyacrylate, (D) polypropylene, (E) polyethylene, (F) polyvinyl chloride, and (G) acrylic-styrenic-copolymer.
