# Supplementary material for: Microplastics in freshwater environments across the high altitude region of Gilgit-Baltistan, Pakistan
Source: Front Toxicol. 2026 Sep 22;8:1947679. doi: 10.3389/ftox.2026.1947679 (PMC13639269; doi:10.3389/ftox.2026.1947679)
Supplement: Supplementary file 2 [file Image2.pdf]

Supplementary Material

(A)

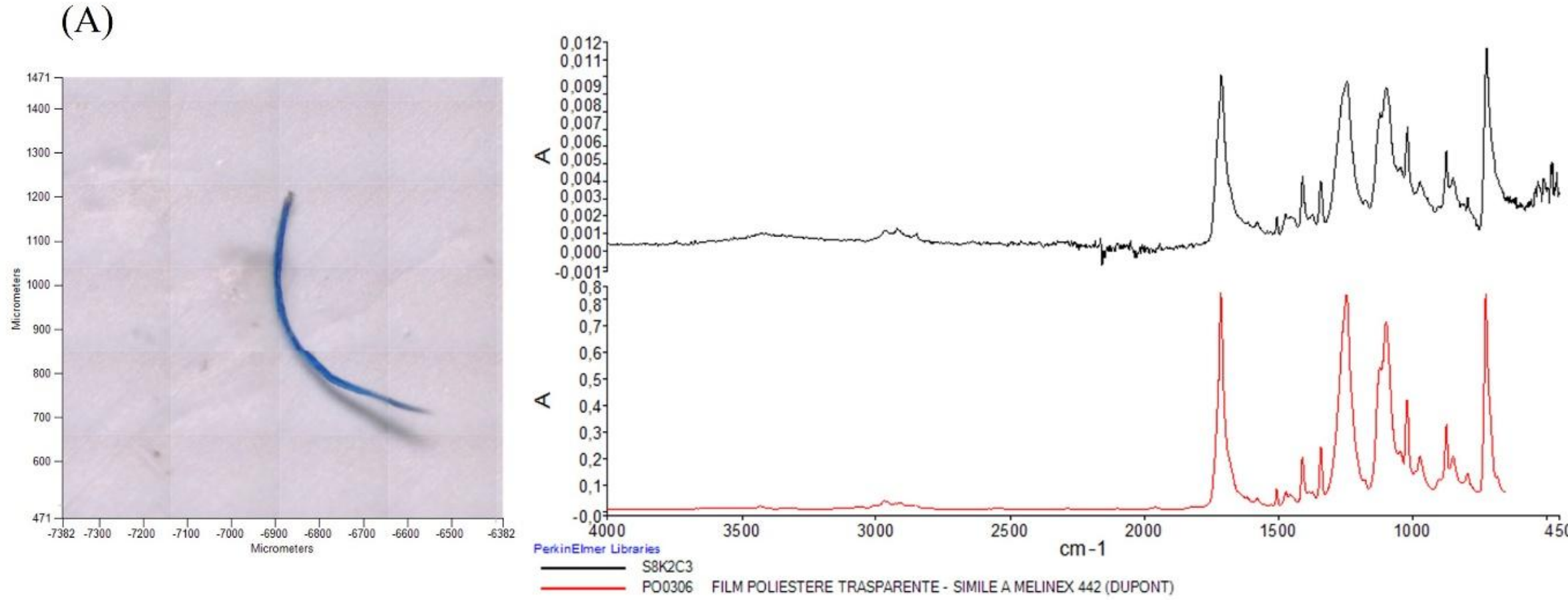

Source Spectra				
**Sample Name**	**Search Score**	**Search Best Hit**	**Search Best Hit Description**	**Search Library**
S8K2C3	0,974421	PO0306	FILM POLIESTERE TRASPARENTE - SIMILE A MELINEX 442 (DUPONT)	POLIMERI

(B)

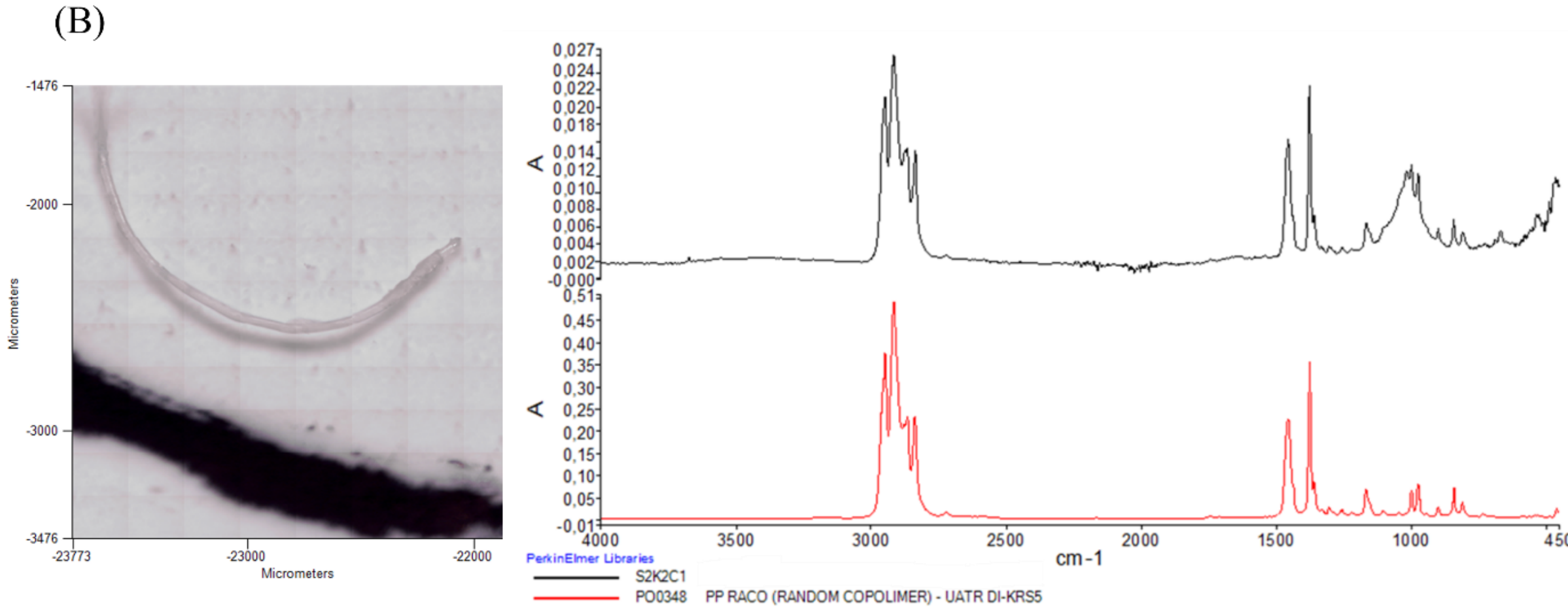

Source Spectra				
**Sample Name**	**Search Score**	**Search Best Hit**	**Search Best Hit Description**	**Search Library**
S2K2C1	0,965822	PO0348	PP RACO (RANDOM COPOLIMER) - UATR DI-KRS5	POLIMERI

(C)

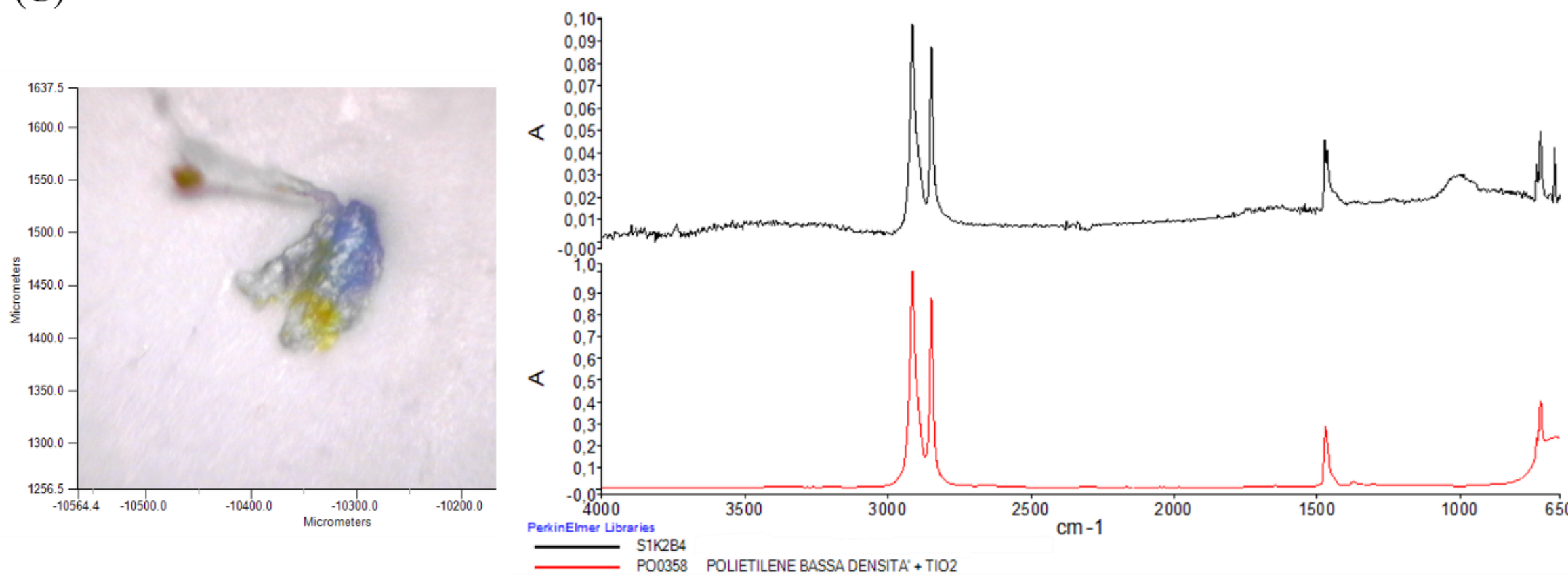

Source Spectra				
**Sample Name**	**Search Score**	**Search Best Hit**	**Search Best Hit Description**	**Search Library**
S1K2B4	0,972333	PO0358	POLIETILENE BASSA DENSITA' + TIO2	PERKIN~1

(D)

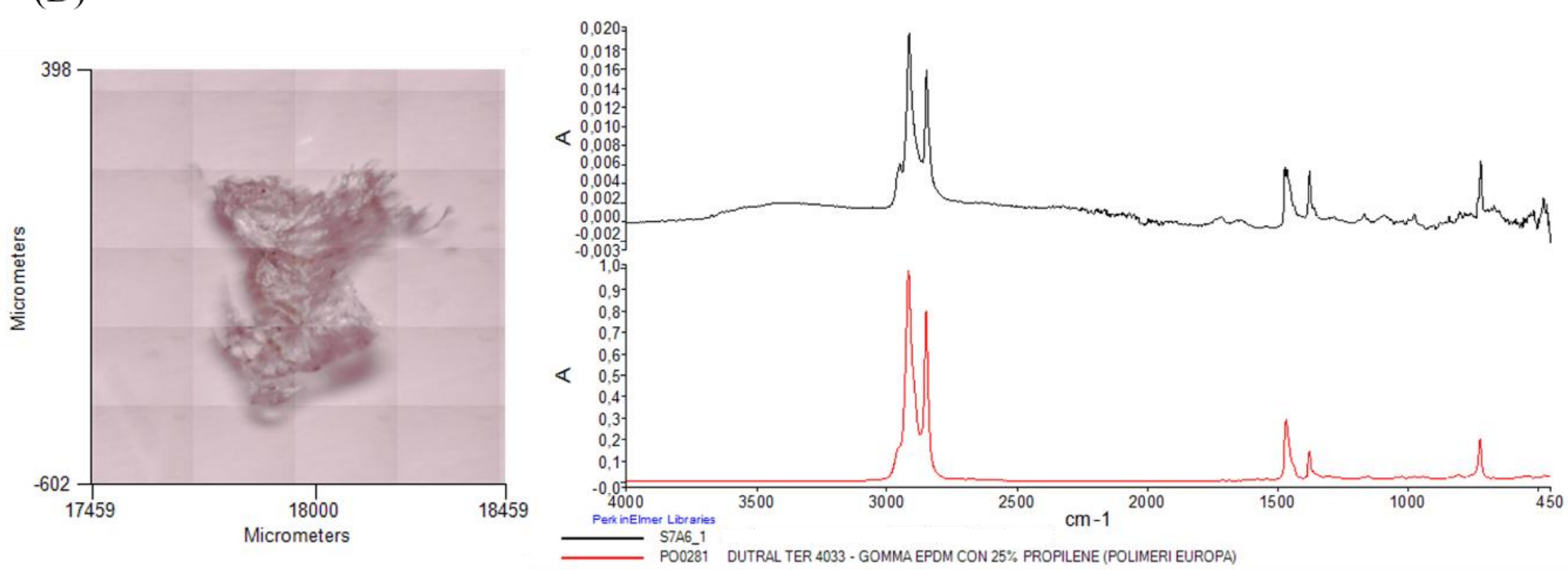

Source Spectra				
**Sample Name**	**Search Score**	**Search Best Hit**	**Search Best Hit Description**	**Search Library**
S7A6_1	0,9327	PO0281	DUTRAL TER 4033 - GOMMA EPDM CON 25% PROPILENE (POLIMERI EUROPA)	PERKIN~1

(E)

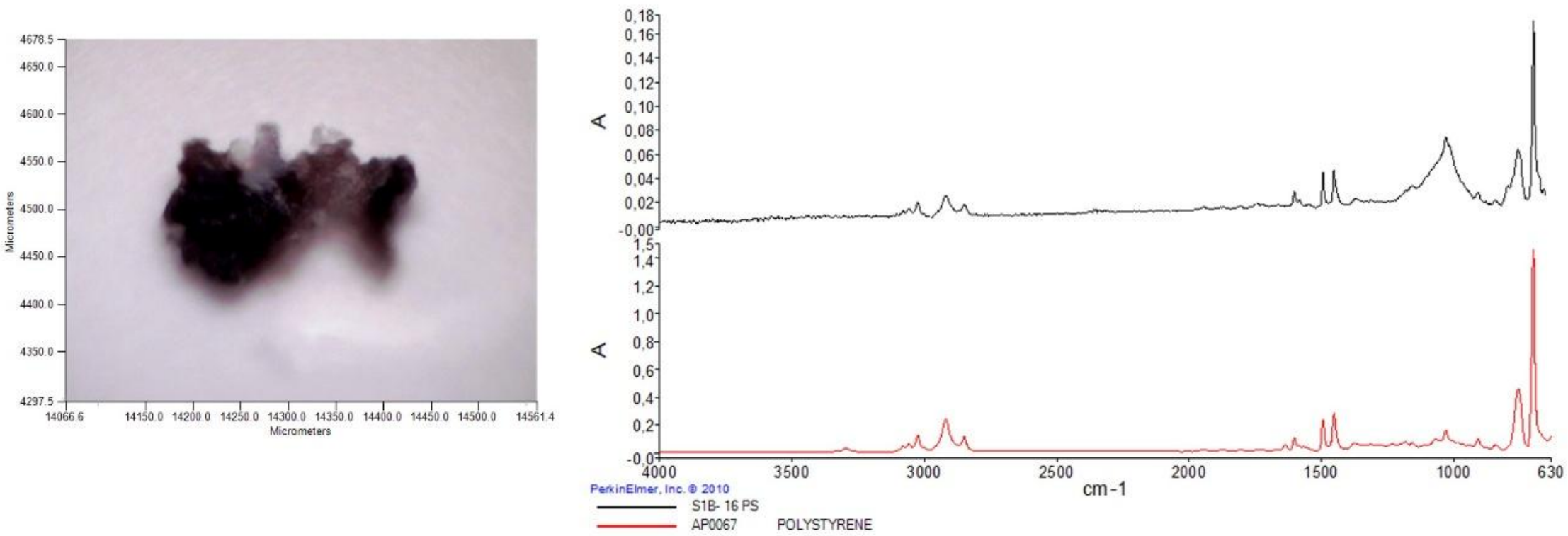

Source Spectra				
**Sample Name**	**Search Score**	**Search Best Hit**	**Search Best Hit Description**	**Search Library**
S1B- 16 PS	0,816252	AP0067	POLYSTYRENE	ATR Polymer Introductory Library

(F)

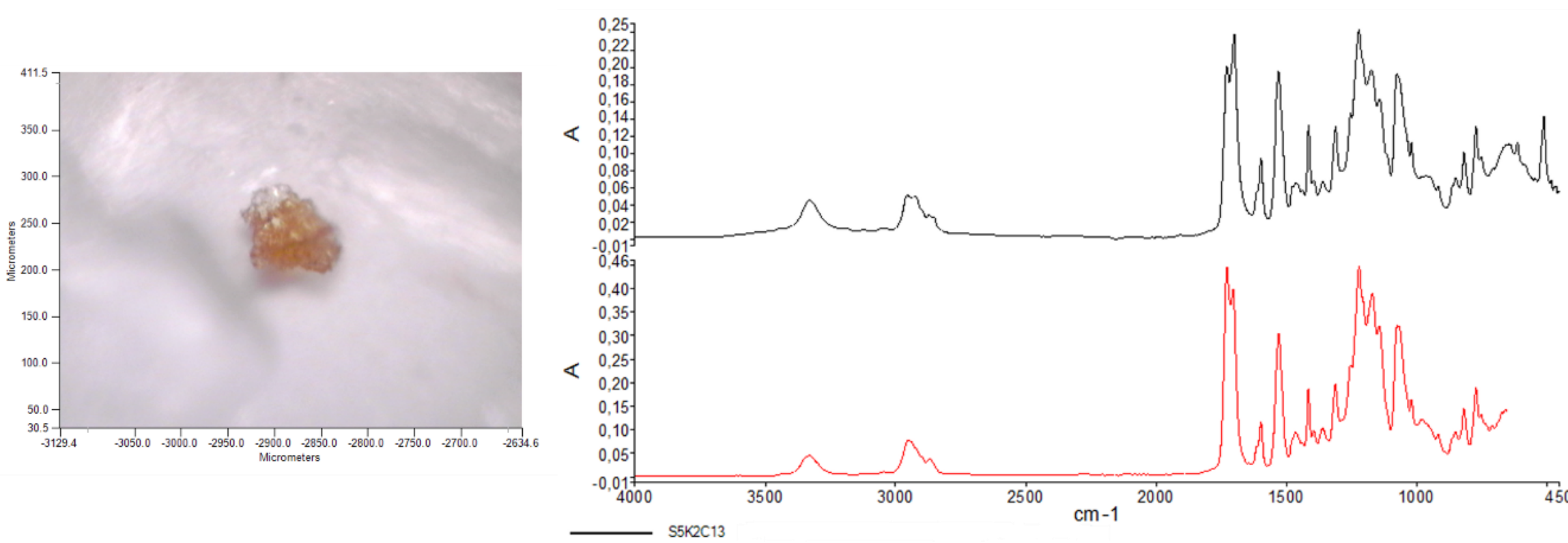

Source Spectra				
**Sample Name**	**Search Score**	**Search Best Hit**	**Search Best Hit Description**	**Search Library**
S5K2C13	0,970367	PO0090	POLIURETANO TERMOPLASTICO BASE ESTERE - ESTANE 5707	PERKIN~1

(G)

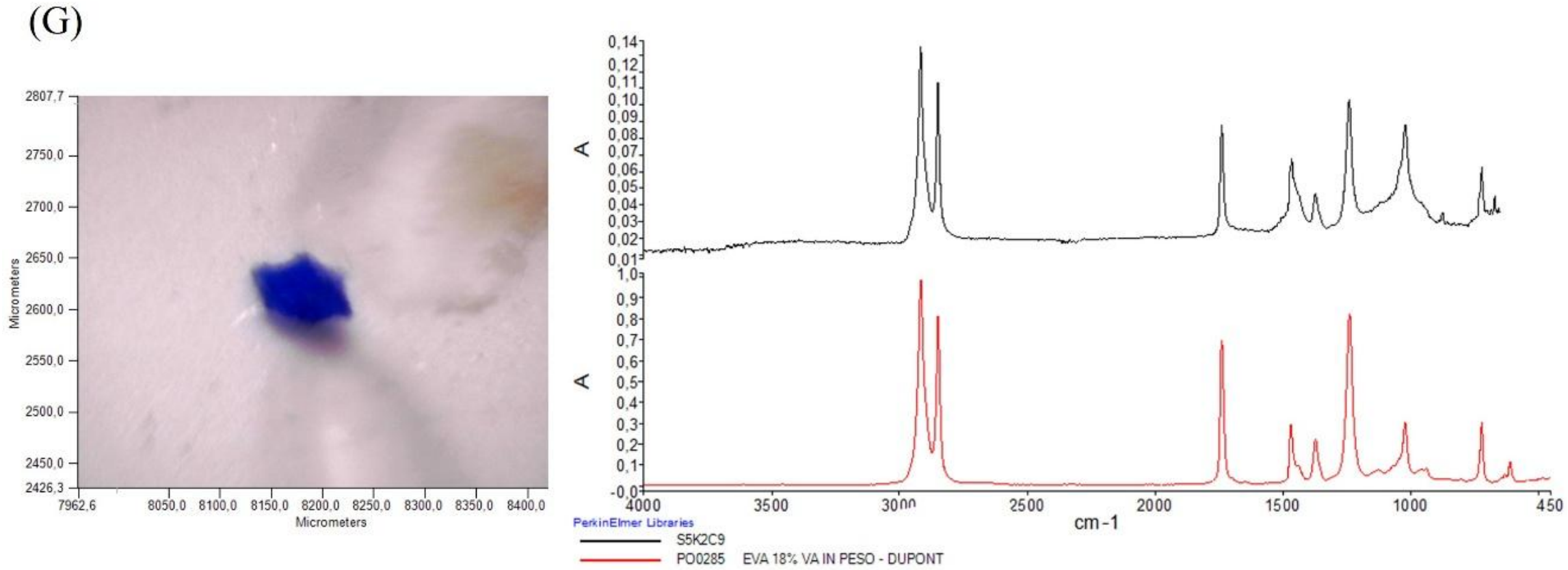

Source Spectra				
**Sample Name**	**Search Score**	**Search Best Hit**	**Search Best Hit Description**	**Search Library**
S5K2C9	0,964667	PO0285	EVA 18% VA IN PESO - DUPONT	POLIMERI

(H)

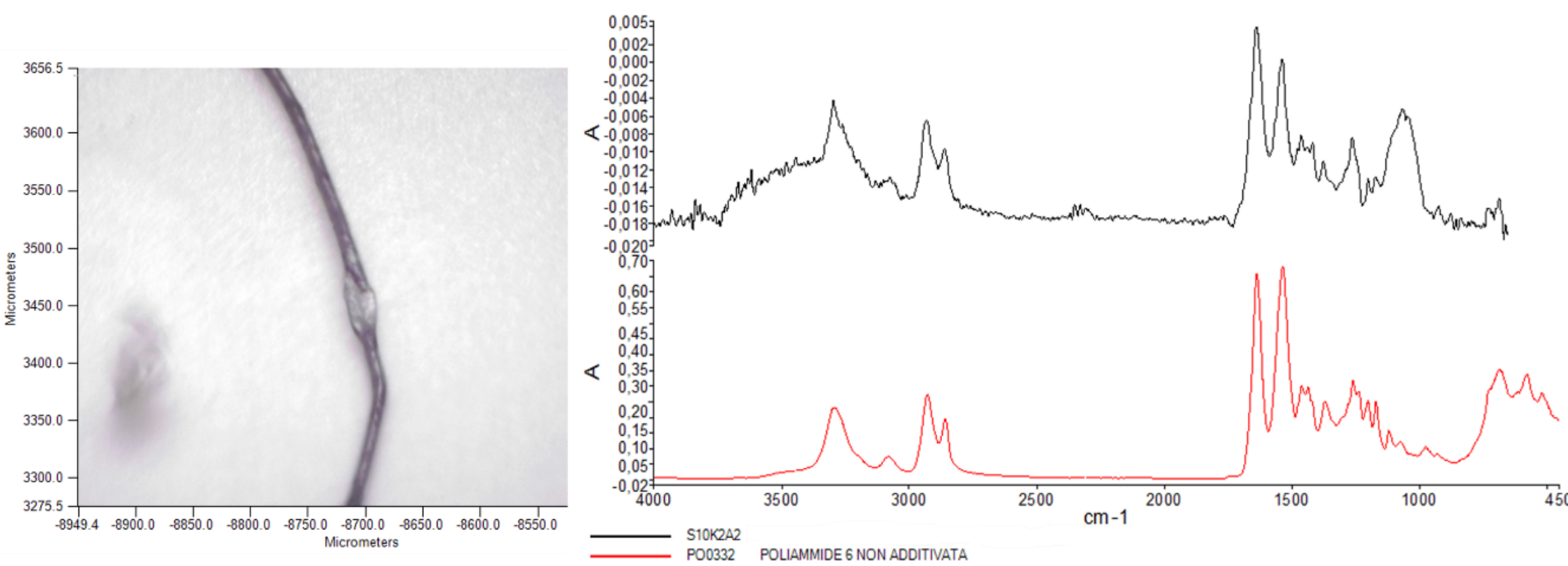

Source Spectra				
**Sample Name**	**Search Score**	**Search Best Hit**	**Search Best Hit Description**	**Search Library**
S10K2A2	0,862628	PO0332	POLIAMMIDE 6 NON ADDITIVATA	POLIMERI

Supplementary Figure 2. Representative µ-FTIR spectra of microplastic particles identified in sediment samples. One representative particle is shown for each of the eight polymer types identified, along with its corresponding µ-FTIR spectrum (black) and the reference spectrum from the spectral library (red). The polymer identification and library match score are reported for each particle. (A) polyester, (B) polypropylene, (C) polyethylene, (D) ethylene propylene diene monomer, (E) polystyrene, (F) polyurethane, (G) ethylene-vinyl acetate, (H) polyamide.
