# Supplementary material for: Microplastics in freshwater environments across the high altitude region of Gilgit-Baltistan, Pakistan
Source: Front Toxicol. 2026 Sep 22;8:1947679. doi: 10.3389/ftox.2026.1947679 (PMC13639269; doi:10.3389/ftox.2026.1947679)
Supplement: Supplementary file 3 [file Image3.pdf]

*Supplementary Material*

(A)

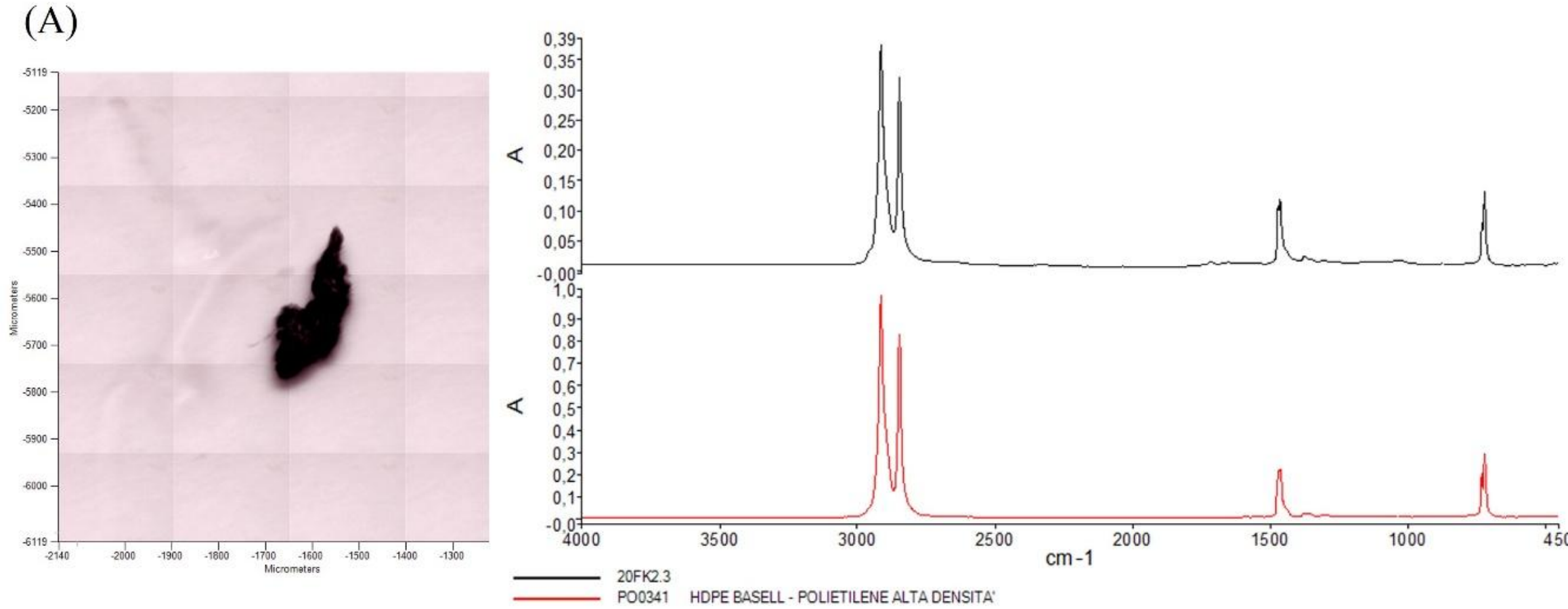


| Source Spectra | | | | |
|---|---|---|---|---|
| **Sample Name** | **Search Score** | **Search Best Hit** | **Search Best Hit Description** | **Search Library** |
| 20FK2.3 | 0,995893 | PO0341 | HDPE BASELL - POLIETILENE ALTA DENSITA' | POLIMERI |

(B)

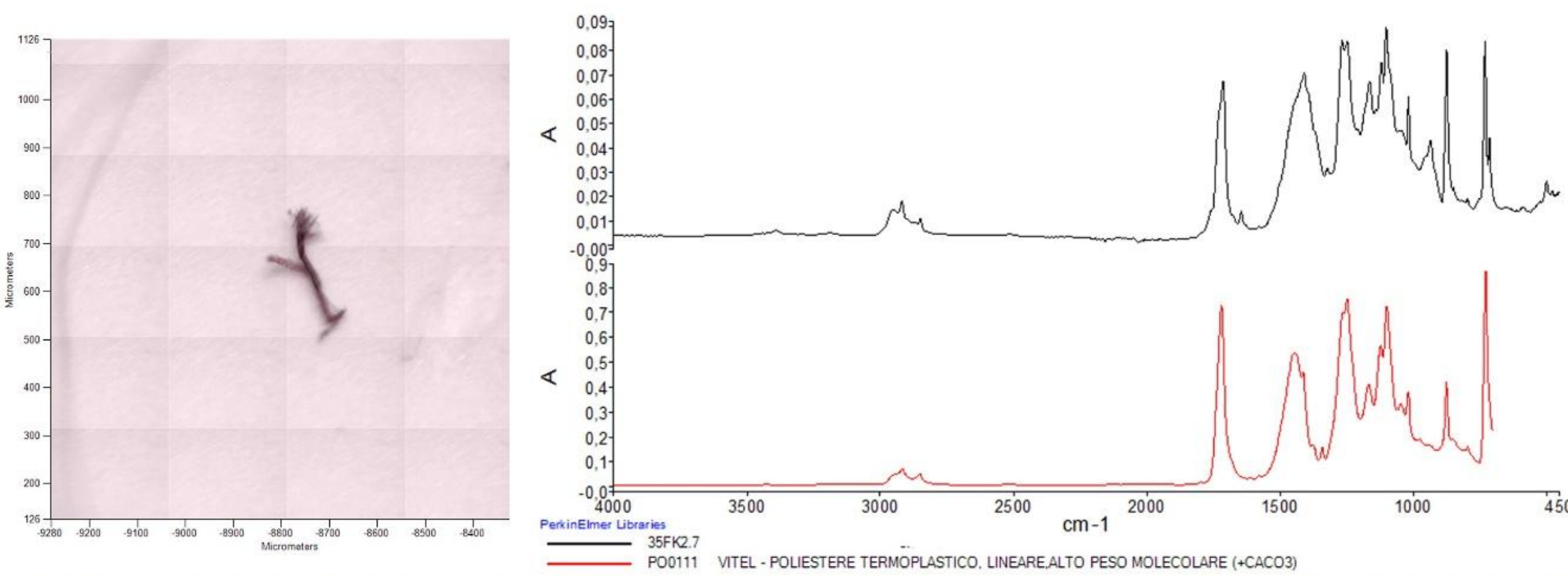


| Source Spectra | | | | |
|---|---|---|---|---|
| **Sample Name** | **Search Score** | **Search Best Hit** | **Search Best Hit Description** | **Search Library** |
| 35FK2.7 | 0,882463 | PO0111 | VITEL - POLIESTERE TERMOPLASTICO, LINEARE,ALTO PESO MOLECOLARE (+CACO3) | PERKIN~1 |

(C)

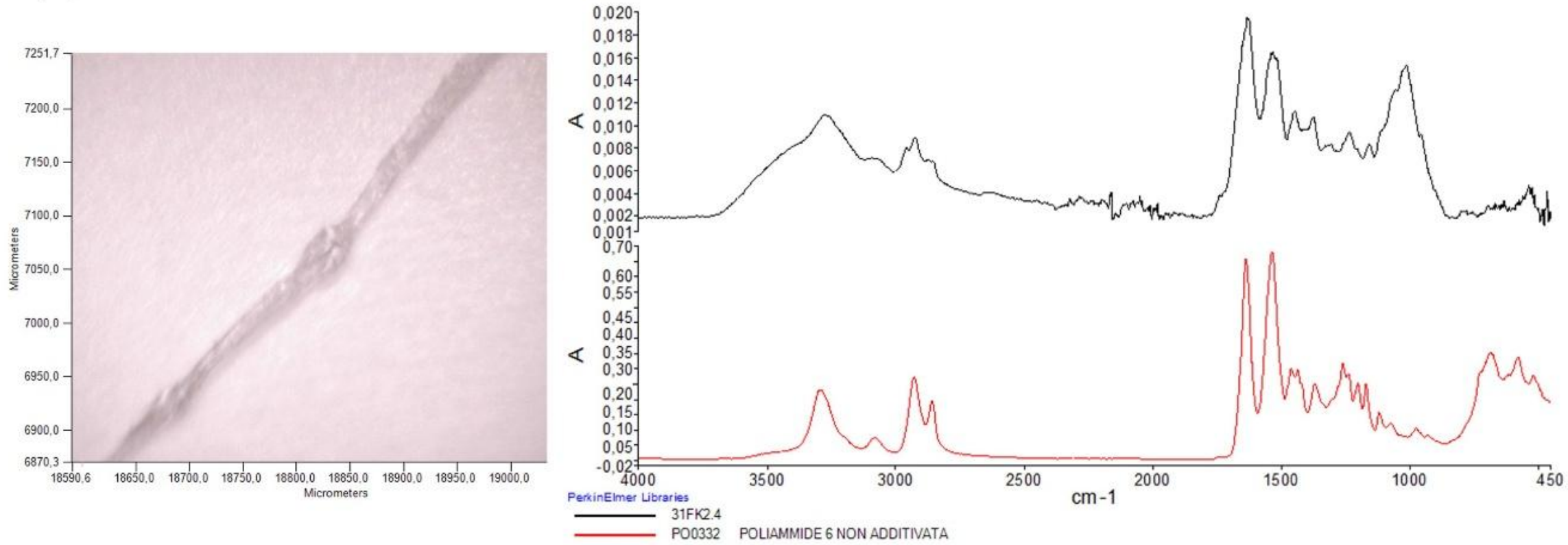


| Source Spectra | | | | |
|---|---|---|---|---|
| **Sample Name** | **Search Score** | **Search Best Hit** | **Search Best Hit Description** | **Search Library** |
| 31FK2.4 | 0,757117 | PO0332 | POLIAMMIDE 6 NON ADDITIVATA | PERKIN~1 |

(D)

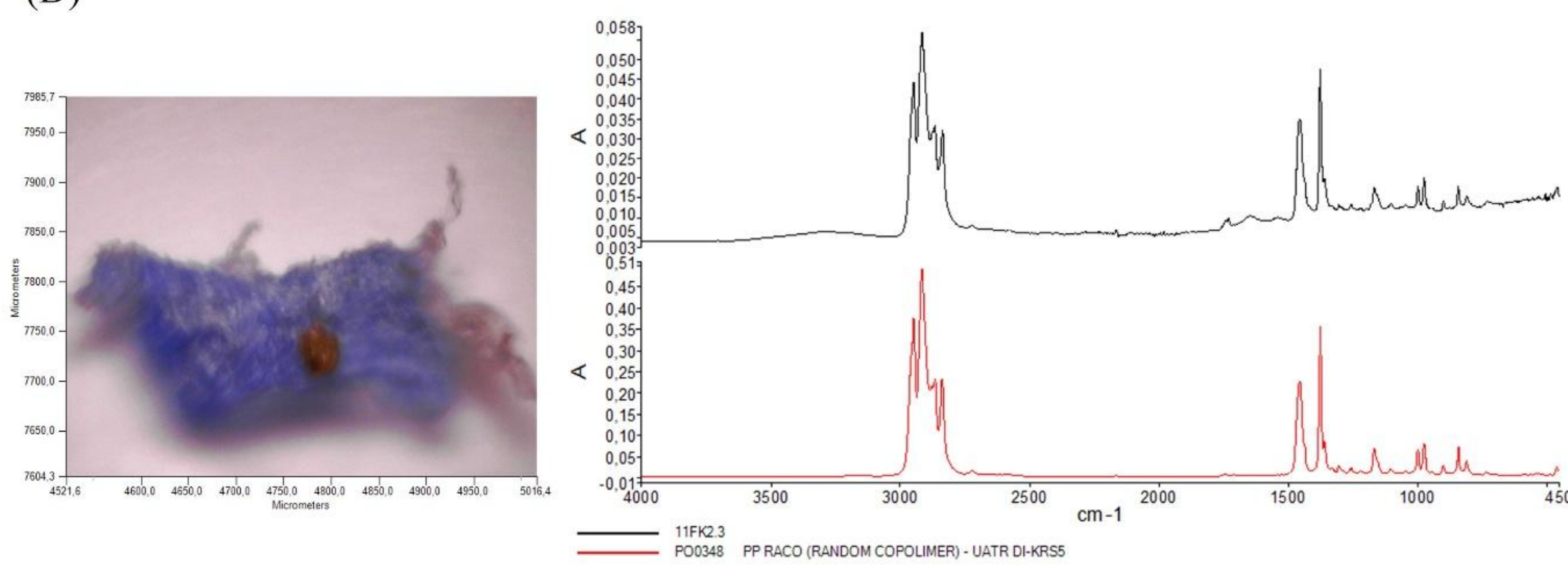


| Source Spectra | | | | |
|---|---|---|---|---|
| **Sample Name** | **Search Score** | **Search Best Hit** | **Search Best Hit Description** | **Search Library** |
| 11FK2.3 | 0,991748 | PO0348 | PP RACO (RANDOM COPOLIMER) - UATR DI-KRS5 | POLIMERI |

(E)

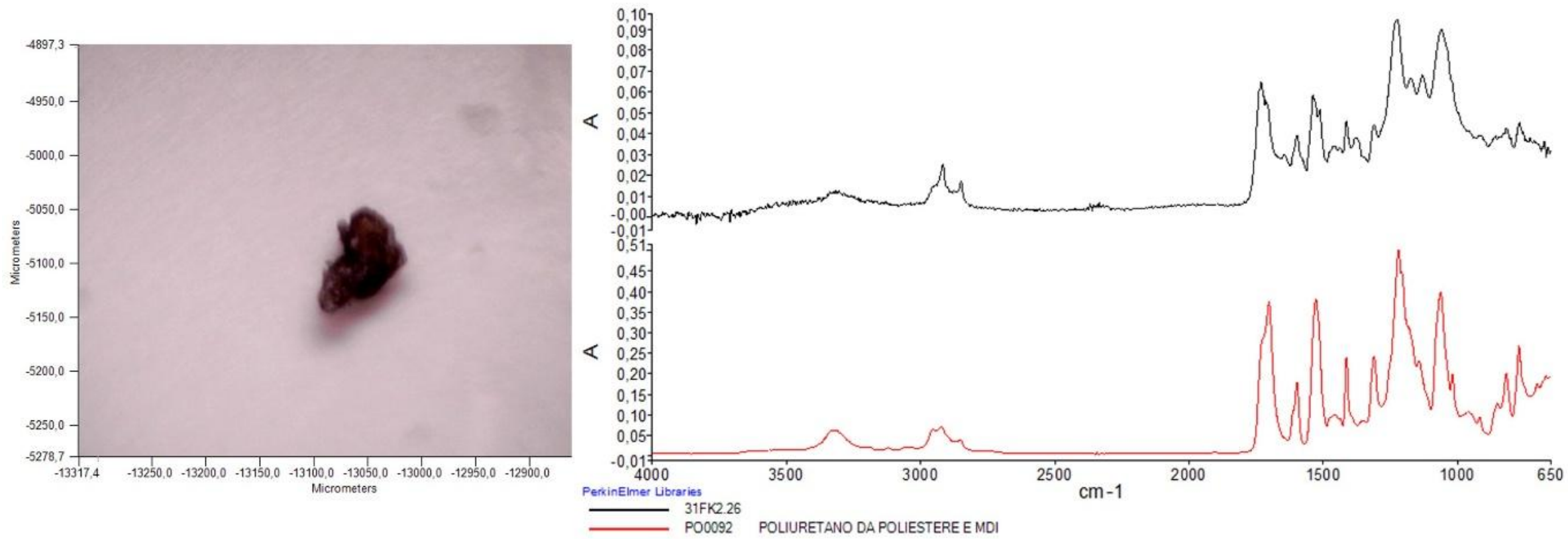


| Source Spectra | | | | |
|---|---|---|---|---|
| Sample Name | Search Score | Search Best Hit | Search Best Hit Description | Search Library |
| 31FK2.26 | 0,78739 | PO0092 | POLIURETANO DA POLIESTERE E MDI | PERKIN~1 |

(F)

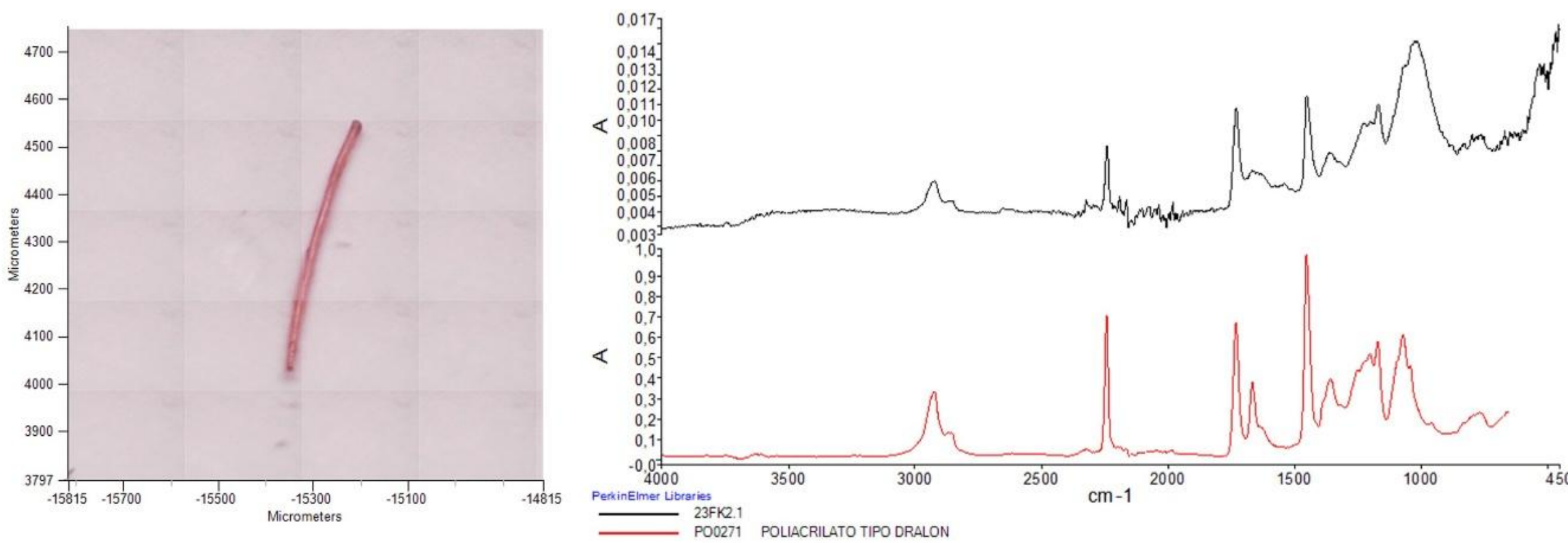


| Source Spectra | | | | |
|---|---|---|---|---|
| Sample Name | Search Score | Search Best Hit | Search Best Hit Description | Search Library |
| 23FK2.1 | 0,818344 | PO0271 | POLIACRILATO TIPO DRALON | POLIMERI |

(G)

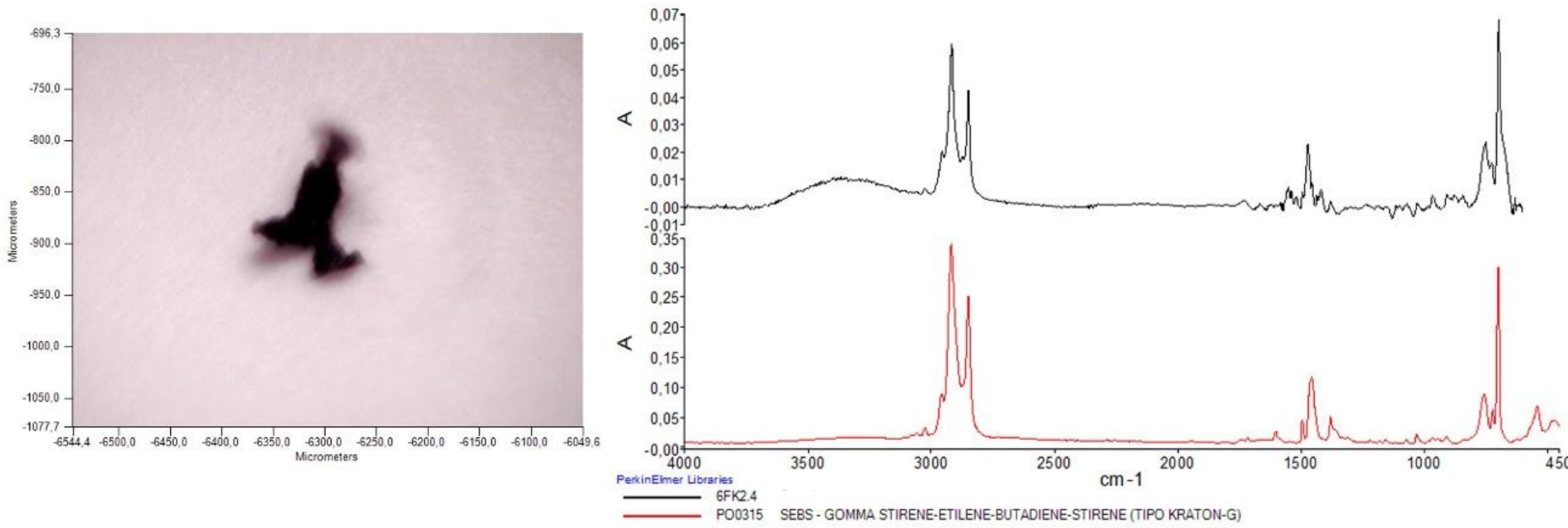


| Source Spectra | | | | |
|---|---|---|---|---|
| **Sample Name** | **Search Score** | **Search Best Hit** | **Search Best Hit Description** | **Search Library** |
| 6FK2.4 | 0,863191 | PO0315 | SEBS - GOMMA STIRENE-ETILENE-BUTADIENE-STIRENE (TIPO KRATON-G) | POLIMERI |

(H)

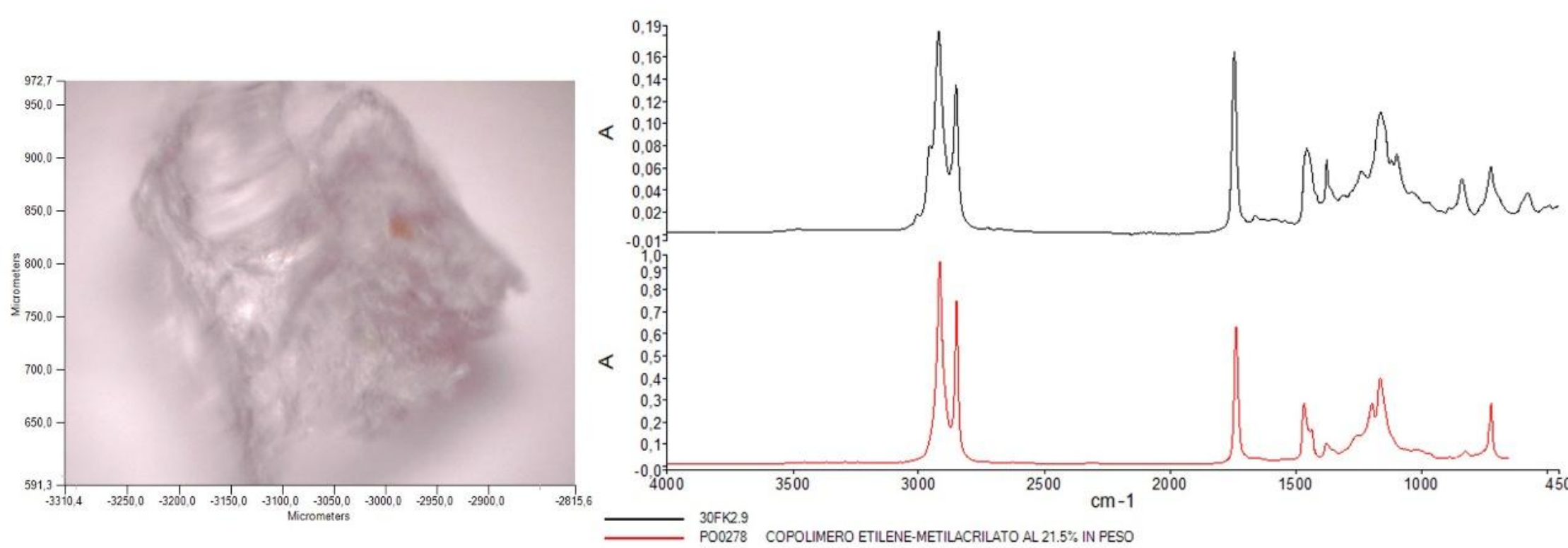


| Source Spectra | | | | |
|---|---|---|---|---|
| **Sample Name** | **Search Score** | **Search Best Hit** | **Search Best Hit Description** | **Search Library** |
| 30FK2.9 | 0,804803 | PO0278 | COPOLIMERO ETILENE-METILACRILATO AL 21.5% IN PESO | PERKIN~1 |

(I)

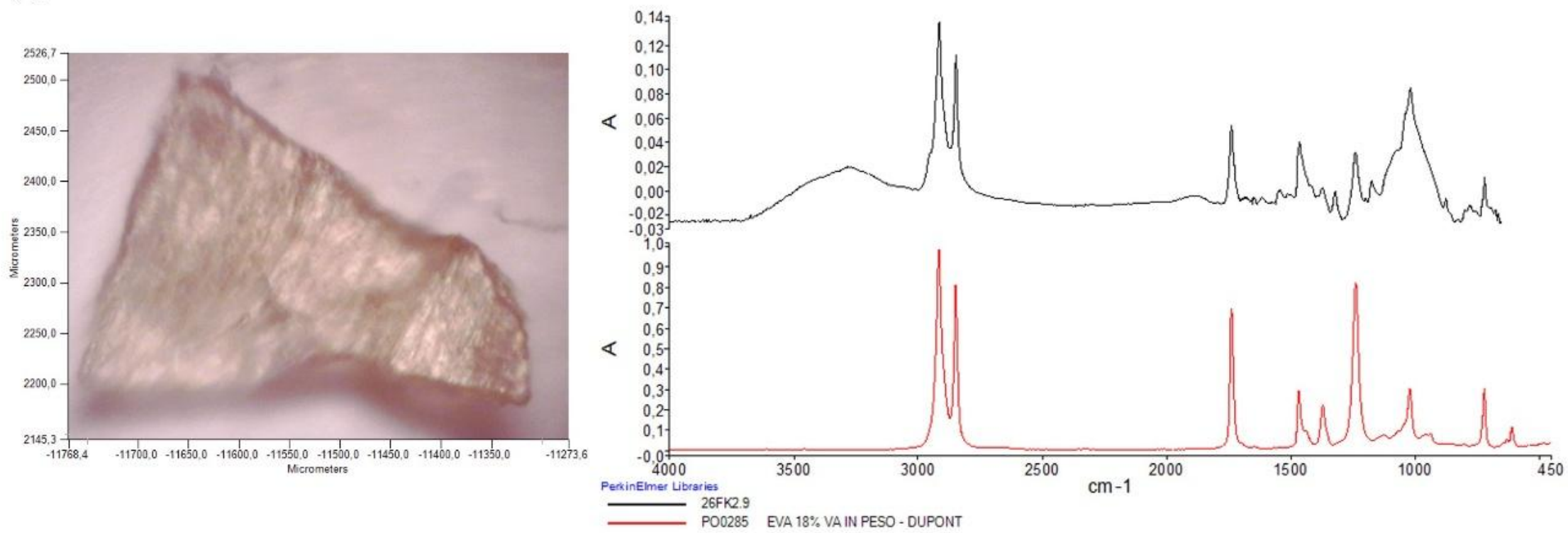


| Source Spectra | | | | |
|---|---|---|---|---|
| **Sample Name** | **Search Score** | **Search Best Hit** | **Search Best Hit Description** | **Search Library** |
| 26FK2.9 | 0,857886 | PO0285 | EVA 18% VA IN PESO - DUPONT | PERKIN~1 |

(L)

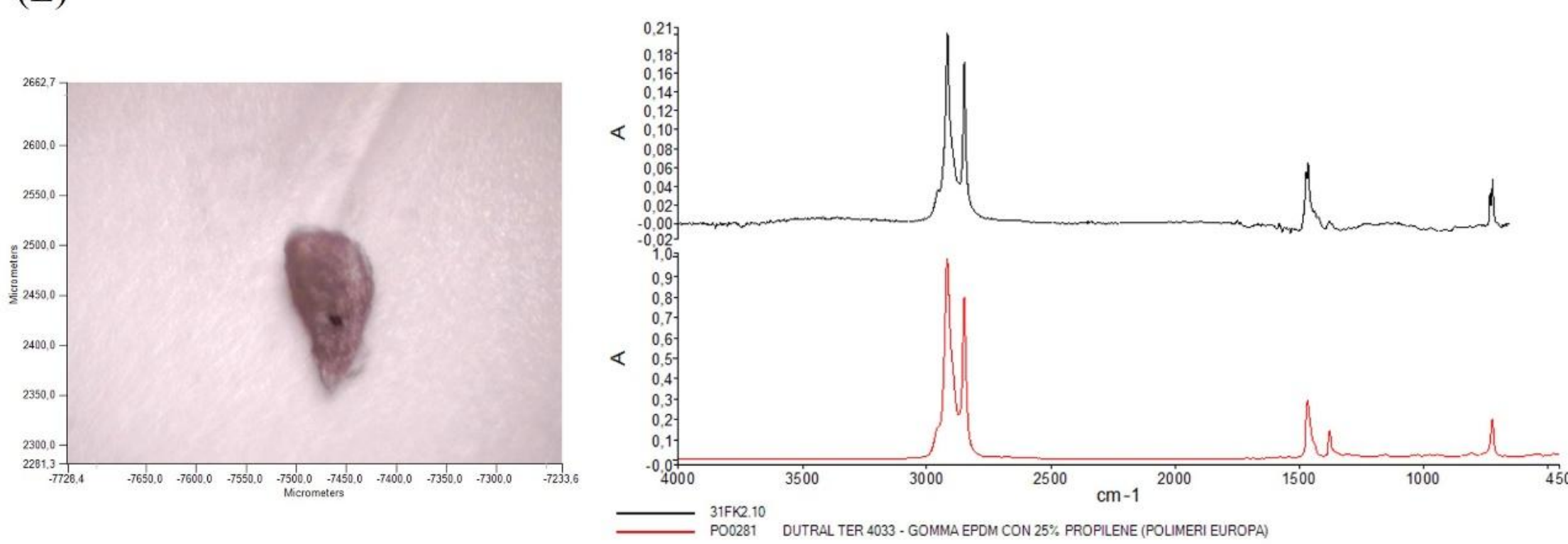


| Source Spectra | | | | |
|---|---|---|---|---|
| **Sample Name** | **Search Score** | **Search Best Hit** | **Search Best Hit Description** | **Search Library** |
| 31FK2.10 | 0,966718 | PO0281 | DUTRAL TER 4033 - GOMMA EPDM CON 25% PROPILENE (POLIMERI EUROPA) | POLIMERI |

(M)

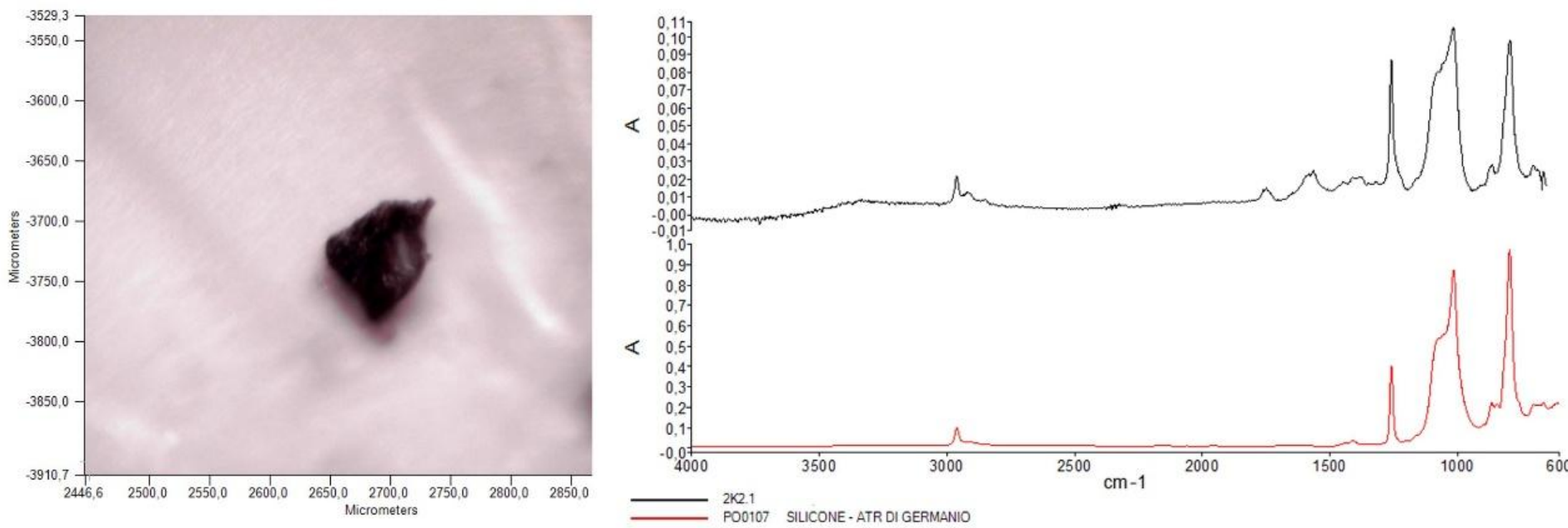


| Source Spectra | | | | |
|---|---|---|---|---|
| Sample Name | Search Score | Search Best Hit | Search Best Hit Description | Search Library |
| 2K2.1 | 0,949989 | PO0107 | SILICONE - ATR DI GERMANIO | POLIMERI |

**Supplementary Figure 3.** Representative μ-FTIR spectra of microplastic particles identified in fish samples. One representative particle is shown for each of the eleven polymer types identified, along with its corresponding μ-FTIR spectrum (black) and the reference spectrum from the spectral library (red). The polymer identification and library match score are reported for each particle. (A) polyethylene, (B) polyester, (C) polyamide, (D) polypropylene, (E) polyurethane, (F) polyacrylate, (G) styrene-ethylene-butylene-styrene, (H) ethylene methyl acrylate, (I) ethylene-vinyl acetate, (L) ethylene propylene diene monomer, (M) silicone.
